# Efficacy and pharmacodynamic effect of anti-CD73 and anti-PD-L1 monoclonal antibodies in combination with cytotoxic therapy: observations from mouse tumor models

**DOI:** 10.1080/15384047.2023.2296048

**Published:** 2024-01-11

**Authors:** Brajesh P. Kaistha, Gozde Kar, Andreas Dannhorn, Amanda Watkins, Grace Opoku-Ansah, Kristina Ilieva, Stefanie Mullins, Judith Anderton, Elena Galvani, Fabien Garcon, Jean-Martin Lapointe, Lee Brown, James Hair, Tim Slidel, Nadia Luheshi, Kelli Ryan, Elizabeth Hardaker, Simon Dovedi, Rakesh Kumar, Robert W. Wilkinson, Scott A. Hammond, Jim Eyles

**Affiliations:** a Oncology R & D, AstraZeneca, Cambridge, UK; b Imaging Sciences, AstraZeneca, Cambridge, UK; c Immunooncology, MorphoSys AG, Planegg, Germany; d Translational Science, F-Star, Cambridge, UK; e Oncology Safety Pathology, AstraZeneca, Cambridge, UK; f Oncology R & D, AstraZeneca, Gaithersburg, MD, USA

**Keywords:** Anti-CD73, Oleclumab, immunotherapy, chemotherapy, adenosine, immune-checkpoint blockade (ICB), radiotherapy, syngeneic tumor models, tumor microenvironment (TME), bulk RNA-sequencing (RNAseq)

## Abstract

CD73 is a cell surface 5’nucleotidase (NT5E) and key node in the catabolic process generating immunosuppressive adenosine in cancer. Using a murine monoclonal antibody surrogate of Oleclumab, we investigated the effect of CD73 inhibition in concert with cytotoxic therapies (chemotherapies as well as fractionated radiotherapy) and PD-L1 blockade. Our results highlight improved survival in syngeneic tumor models of colorectal cancer (CT26 and MC38) and sarcoma (MCA205). This therapeutic outcome was in part driven by cytotoxic CD8 T-cells, as evidenced by the detrimental effect of CD8 depleting antibody treatment of MCA205 tumor bearing mice treated with anti-CD73, anti-PD-L1 and 5-Fluorouracil+Oxaliplatin (5FU+OHP). We hypothesize that the improved responses are tumor microenvironment (TME)-driven, as suggested by the lack of anti-CD73 enhanced cytopathic effects mediated by 5FU+OHP on cell lines *in vitro*. Pharmacodynamic analysis, using imaging mass cytometry and RNA-sequencing, revealed noteworthy changes in specific cell populations like cytotoxic T cells, B cells and NK cells in the CT26 TME. Transcriptomic analysis highlighted treatment-related modulation of gene profiles associated with an immune response, NK and T-cell activation, T cell receptor signaling and interferon (types 1 & 2) pathways. Inclusion of comparator groups representing the various components of the combination allowed deconvolution of contribution of the individual therapeutic elements; highlighting specific effects mediated by the anti-CD73 antibody with respect to immune-cell representation, chemotaxis and myeloid biology. These pre-clinical data reflect complementarity of adenosine blockade with cytotoxic therapy, and T-cell checkpoint inhibition, and provides new mechanistic insights in support of combination therapy.

## Introduction

Extracellular adenosine has emerged as an important regulator of immunological processes within the tumor microenvironment and is thought to diminish the effectiveness of T-cell checkpoint inhibiting drugs as well as anti-tumor immunity in general.^1–3^ Adenosine triphosphate (ATP), released from necrotic or damaged cells, is hydrolyzed to adenosine by the sequential action of two ectonucleosidases working in tandem: CD39 (ENTPD1) and CD73 (NT5E). The resultant adenosine acts as a readily diffusible immunosuppressive ‘smog’, and it is likely that cytotoxic agents and radiotherapy (RTx) exacerbate this process. To this point, enhanced anti-tumor activity is observed in preclinical models when RTx is combined with a CD73 inhibiting antibody treatment.^4,5^ Those studies highlighted an important role for radiation induced type-1 interferons in driving elevated tumoral cDC1 infiltration levels and a beneficial effect of CD73 inhibition on this biomarker when radiation activated type-1 interferon levels were suboptimal.^4^ Despite new information on the role of CD73 in the context of radiation-based standard-of-care, relatively little is known about the effects of adenosine pathway inhibiting drugs within the context of chemotherapy treatment regimens, even though a growing body of evidence points to an immunomodulatory axis for these agents.^6^ Inclusion of T-cell checkpoint inhibitors within this paradigm provides further scope for enhanced cell-mediated activity; by counteracting adaptive immune-resistance and unfettering anti-tumor immunity.

Oleclumab, a CD73 inhibiting human monoclonal IgG1-TM antibody^7^is currently in phase 2/3 of clinical development in combination with Durvalumab for treatment of patients with various solid tumors. Data from a Phase II platform study of Durvalumab in combination with Oleclumab in patients with unresectable stage III non-small-cell lung cancer (NSCLC), who had not progressed after prior chemoradiotherapy, highlighted significant benefit of the Oleclumab component.^8^ A phase 3 clinical trial is now underway in the same patient population.^9^

Using mouse models of cancer and a murine surrogate of Oleclumab (hereafter called aCD73), we explored the effects of CD73 inhibition in combination with chemotherapies and PD-L1 blockade. As a corollary, we explored whether the same approach can be applied to enhance the effects of fractionated RTx. We found these combinations were highly effective in terms of improved tumor growth inhibition and overall survival benefit. Transcriptomic-based pharmacodynamic assessments highlighted increased abundances of cytotoxic lymphocytes and immune-supportive myeloid populations in the tumor. Profiling of treatment groups representing the various components of the combination allowed deconvolution of contributing individual therapeutic components; highlighting interesting effects conferred by CD73 inhibition in the context of the combined chemotherapy and PD-L1 blockade. These preliminary data are encouraging and translationally informative, with respect to the use of CD73 inhibition within the context of T-cell checkpoint inhibitor augmented ‘standard-of-care’ cytotoxic treatment regimens.

## Results

### Combined aCD73, aPD-L1 and 5FU+OHP treatment leads to enhanced complete responses in syngeneic mouse models

**Figure 1.**
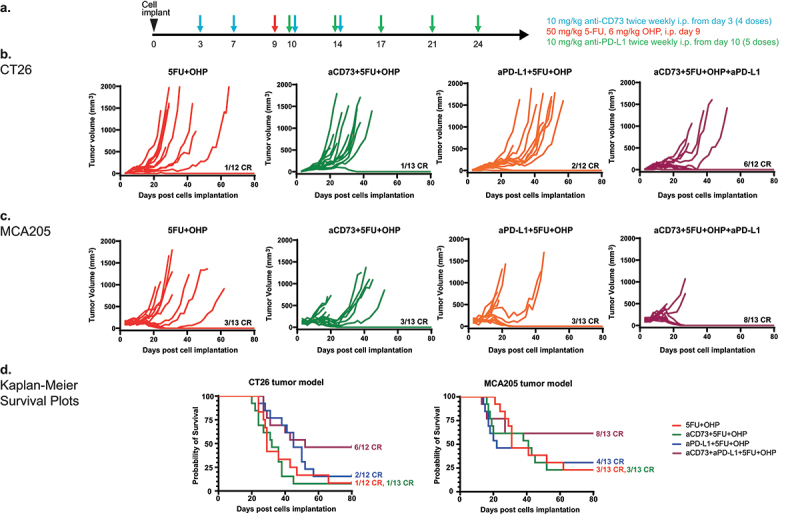
Combined aCD73, aPD-L1 and 5FU+OHP treatment leads to enhanced complete responses in syngeneic mouse models. (a) schematic of the experimental design. (b) BALB/c (CT26 cells in PBS) and (c) C57BL/6J mice (MCA205 cells in 50% matrigel + PBS) were implanted with 5e5 cells in the right flank and treated as shown in schematic. Growth curves are plotted from calliper measurements done thrice weekly. Addition of aCD73 and aPD-L1 to 5FU+OHP lead to significant increase in the number of complete responders (CR) in each model system- 50% in CT26 (*p*=.005 vs 5FU+OHP treated group) and 61.5% in MCA205 (*p*=.008 vs 5FU+OHP treated group); Kruskal Wallis test. (d) Kaplan Meier survival plots showing that addition of aCD73 and aPD-L1 to 5FU+OHP significantly increased survival in the two tumor models (CT26- left panel and MCA205- right panel).

Combined treatment with aCD73 and aPD-L1 antibodies, in addition to 5FU+OHP resulted in enhanced efficacy and complete responses (Figure 1d) in two mouse syngeneic models of cancer- CT26 (p = .005 vs 5FU+OHP alone) and MCA205 (p = .008 vs 5FU+OHP alone) as shown in Figure 1(b,c) (Kruskal-Wallis test). Treatment of CT26 or MCA205 tumor bearing mice with aCD73 as a monotherapy resulted in negligible benefit in terms of tumor growth inhibition. aPD-L1 treatment retarded tumor growth rates in a proportion of mice bearing CT26 tumors (Supplementary Figure S1A and S1C) but displayed minimal activity in the MCA205 model as shown in Supplemental Figure S1B and S1D). 5FU+OHP chemotherapy suppressed tumor growth rates in both the models but combining 5FU+OHP with either immune-oncology (IO) agents (aCD73 or aPD-L1) exerted minimal further uplift in efficacy (Supplementary Figure S1C and S1D). A combination treatment utilizing aCD73 and aPD-L1 antibodies (aCD73+aPD-L1) afforded similar levels of tumor growth control to anti-PD-L1 alone, highlighting the importance of the chemotherapy component within the chemotherapy+IO combination (Figure S1A and S1B).

### Effect of aCD73 on chemotherapy-induced cytotoxicity in cell culture

In parallel, we hypothesized whether the anti-tumor effects of aCD73 *in vivo* occurred through the cells in the tumor microenvironment (TME) or directly on the tumor cells. To test this hypothesis we investigated whether blocking CD73 with aCD73 could enhance the direct cytotoxic effect of 5FU+OHP on the cell lines themselves. However, we found no evidence that aCD73 improved 5FU+OHP cytotoxicity in these *in vitro* settings (Supplementary Figure S2). We also tested whether the cytotoxic effects of docetaxel on cultured CT26 cells was influenced by the presence of aCD73, and were unable to detect any changes with the addition of the antibody.

### CD8 cell depletion compromises efficacy of the quadruple combination (aCD73+aPD-L1+5FU+OHP) in MCA205 tumor bearing mice

**Figure 2.**
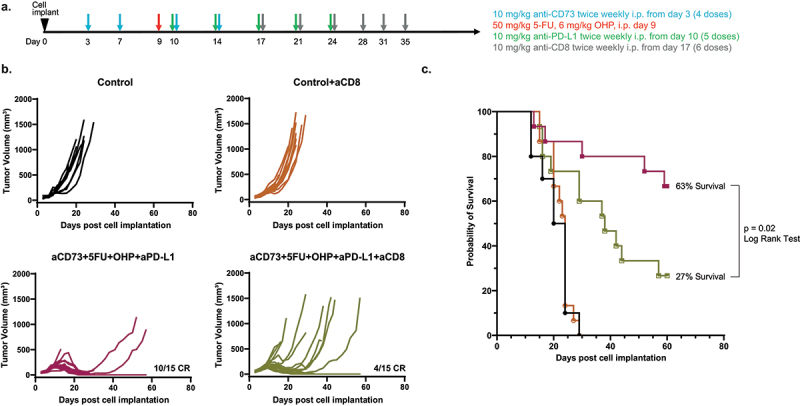
CD8 depletion leads to loss of efficacy seen in combined aCD73, aPD-L1 and 5FU+OHP treatment in syngeneic mouse model MCA205. (a) schematic of the experimental design. (b) growth curves in C57BL/6J mice of MCA205 tumor model (in 50% matrigel + PBS 5e5 cells) were plotted from calliper measurements done thrice weekly. Selective depletion of CD8 T cells resulted in decreased efficacy to the combination treatment leading to significantly shorter survival span (Kaplan Meier Plot, log rank test, *p*= .02) compared to the those where no CD8 cell depletion was carried out as shown in (c).

To test the hypothesis whether CD8 T-cells played a part in the enhanced efficacy of the combination treatment, we selectively depleted CD8 T cells in the MCA205 model, using IP injection of clone 53–6.7 from the 17th day post tumor implantation (schematic Figure 2a). This experiment confirmed significantly diminished tumor control and long-term survival (by > 50%) in the combination (5FU+OHP+aCD73+aPD-L1) treated group (Log rank test, p = .026, Figure 2c). These CD8 depletion data suggest cell-mediated immunity plays an essential role in the observed activity of the chemotherapy-IO combination. However the results do not rule out contributions by other immune-system components to immune-mediated tumor growth control. Consistent with this, flow cytometric analysis of digested CT26 tumors highlighted mice treated with aCD73 and aPD-L1 plus 5FU+OHP indeed had increased frequencies of IFNγ secreting CD8 and NK cells (Supplementary Figure S3A and S3B). Similarly, “conventional” cytotoxic or cytostatic effects mediated by 5FU+OHP likely play some role in overall combination treatment effect.

### ‘Early’ pharmacodynamic effect of aCD73 + 5FU+OHP with respect to CD73 expression, adenosine pathway metabolites and stromal cell populations in the murine CT26 tumor model

**Figure 3.**
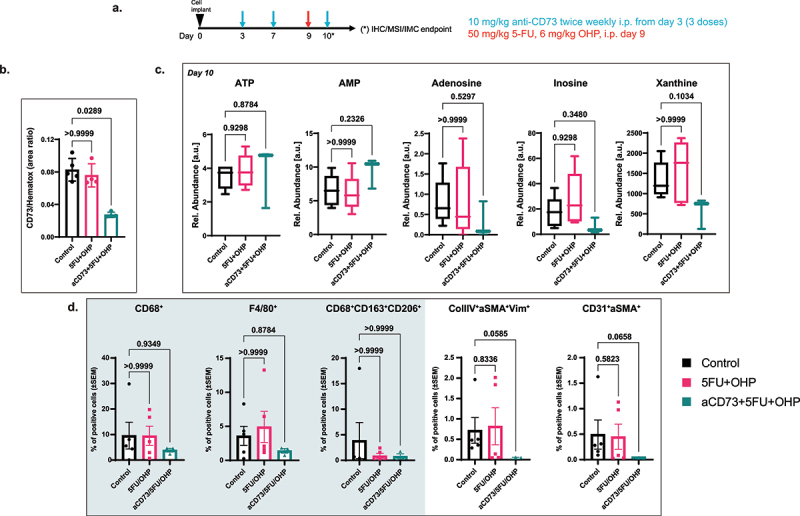
IHC and MSI analysis confirms target (CD73) engagement and adenosine pathway modulation by addition of aCD73 to 5FU+OHP. (a) schematic of the experimental design. (b) Immunohistochemistry analysis revealed significantly lower levels of surface bound CD73 protein in the aCD73 to 5FU+OHP combination group. Results are expressed as area ratio of specific stain to background stain (hematoxylin). (c) mass spectrometry Imaging showed a notable trend in adenosine, inosine and xanthine suppression as early PD biomarkers in the CT26 tumors from mice treated with anti-CD73 containing triple combination group. Results are reported as relative abundance in arbitrary units. (d) Imaging mass cytometry highlighted that CT26 tumors from aCD73+5FU+OHP combination treated mice presented lower frequencies of cells expressing macrophage markers like CD68, F4/80 as well as suppressive tumor associated macrophage markers like CD163 and CD206 [left shaded panel] as well as markers known to associate with cancer-associated fibroblasts like collagen-iv, alpha smooth muscle actin, vimentin along with CD31. Results are expressed as mean ± SEM of %positive cells. Kruskal-Wallis test followed by Dunn’s correction for multiple comparison was used for statistics.

Pharmacodynamic activity of the aCD73 and aPD-L1 plus 5FU+OHP chemotherapy combination, was evaluated using immunohistochemistry (IHC) and imaging mass cytometry (IMC) to understand the early changes mediated by CD73 blockade. As anticipated, the murine surrogate antibody of Oleclumab significantly lowered CD73 levels in CT26 tumors (Figure 3b, Kruskal-Wallis test followed by Dunn’s correction for multiple comparison.). The IHC-based detection of CD73 employed an antibody that did not compete with the murine surrogate antibody of Oleclumab; therefore, the lower levels of CD73 protein were consistent with Oleclumab’s known ability to internalize CD73.^7^ IMC highlighted that CT26 tumors from aCD73 treated mice tended to have lower frequencies of cells expressing markers known to associate with suppressive tumor associated macrophages (Figure 3d shaded panel) and with cancer associated fibroblasts (Figure 3d). Mass Spectroscopy Imaging (MSI) revealed a notable trend for adenosine, inosine and xanthine suppression in CT26 tumors from mice treated with anti-CD73 (Figure 3c and Supplementary Figure S4B). Conversely adenosine monophosphate (AMP), the main substrate of CD73, appeared elevated relative to other treatment groups, in tumor tissue from mice treated with chemotherapy and the aCD73 combination. These pharmacodynamic data are consistent with Oleculmab’s proposed mechanism of action; in terms of CD73 targeting, enzymatic inhibition and adenosine pathway modulation.

### Pharmacodynamic effect of aCD73+aPD-L1+5FU+OHP    with respect to CT26 transcriptome

To gain a deeper understanding of the mechanistic basis for the enhanced activity of the combination (5FU+OHP+aCD73+aPD-L1) group, we used RNAseq to explore changes within the CT26 transcriptome. Using DEseq2 and fGSEA packages, we identified minimal changes in the CT26 transcriptome following aCD73 or aPD-L1 treatment as monotherapies (Supplementary Table S2). 5FU+OHP, on the other hand, was highly perturbing; causing significant upregulation of 277 genes and downregulation of a further 158 transcripts, relative to control tumors. Gene Ontology (GO BP) base pair enrichments and KEGG pathway analysis of these data highlighted significant effects on genes associated with immune response, leukocyte, NK and T-cell activation, T cell receptor signaling, and Interferon (types 1 & 2) production (adjusted-pval <0.05). Individual genes upregulated in line with this included: CCL3, CCL4, CCL8, CCL17, CXCL10, GDF15, CD8a, IFNγ, Perforin, Granzyme, LAG-3 and PD-1. CXCL2 expression was reduced (Supplementary Figure S7). These RNAseq data highlight that 5FU+OHP treatment exerts significant perturbance of genes associated with immune-functionality in the murine CT26 mouse model of cancer.

In contrast to the minor transcriptomic changes observed with the constituent monotherapy treatments, the combination of aCD73 and aPD-L1 resulted in 1236 differentially expressed genes. Most significant and wide-ranging transcriptomic changes were achieved with the 5FU+OHP+aCD73+aPD-L1 combination; causing 1490 genes to be upregulated and 128 down regulated in comparison to untreated tumors (Supplementary Table S2). KEGG pathway and Gene Ontology enrichments analysis highlighted chemotactic mobilization, T cell activation, T cell receptor signaling, Th1 and Th2 cell differentiation and Natural killer cell mediated cytotoxicity. The most affected immune-related genes are listed in supplementary Figure S6. There were several notable additions, to the genes upregulated by 5FU+OHP treatment, including (but not limited to): CD38, CD39, CXCL1, CXCL3, CXCL5, CD163, CTLA4, CXCR3, Granzyme A, ICAM1, IL6, P2RY1, TNF-a, IL2A, IL1A, ALOX15, SLC7a2, EAR2, HAVC2.

Inclusion of treatment groups representing the various components of the combination allowed deconvolution of the contribution of the individual therapeutic components (Figures (4a,b) and S6, S7, ST2). This analysis highlighted a critical role for 5FU+OHP in driving interferon pathway (types 1 and 2) activation alongside T-cell and NK cell activation, cytotoxic activity and IL2/STAT5 pathway signaling. The effects of 5FU+OHP inclusion were largely differentiated from those mediated by addition of either of the IO drug components to the treatment, as evidenced the 360 genes uniquely upregulated and 122 downregulated by the 5FU+OHP component. Including 5FU+OHP within the combined aCD73+aPD-L1 treatment approach served to elevate expression of key immune-related genes such as Interferon-gamma, TRIM6, CCL17, Granzyme B, GDF15, perforin and LAG3. Conversely, IL10, IL1B, CXCL2, S100A8 were downregulated when 5FU+OHP was included.
**Figure 4B.**
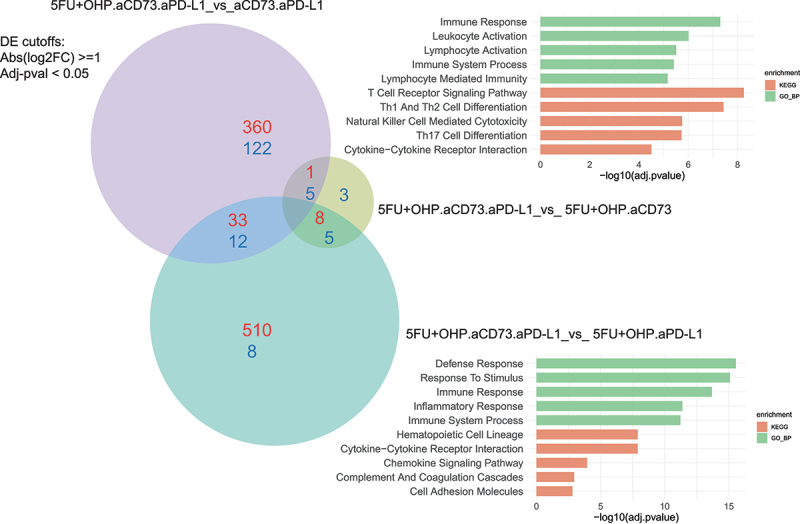
Transcriptome-based deconvolution of key effects mediated by combination. Deconvolution of RNAseq analysis revealed the key pathways affected by addition of aCD73 and 5FU+OHP were the immune response activation pathways. Enriched pathways, KEGG (Kyoto Encyclopedia of Genes and Genomes) pathway enrichment KEGG, green) and gene Ontology>biological process (GO BP, red), for the up-regulated genes are shown (DE cut-off values used were log2FC ≥1 and adj-pval < 0.05).

Adding CD73 blockade to the combination of 5FU+OHP and aPD-L1 upregulated 510 genes and downregulated 8, that were not modulated within other iterations of the combination as shown in Figure 4a. Notably influenced genes included CXCR3, H2-AB1, ITGAE, CXCL3, MGL2, CXCR5, CD4 and CYBB. This also drove even higher expression of CCL17, a major tumor infiltrating lymphocyte (TIL) attracting chemokine, and CCL24 a chemokine known to preferentially chemoattract M1 macrophages.^10^ These data highlight a novel and impactful role for adenosine pathway inhibition within chemotherapy/checkpoint inhibitor combinations. Particularly with respect to individual genes and signatures linked to myeloid and B cell biology. CD38 and P2Y1, two genes known to be linked to the adenosine pathway itself, were also significantly upregulated when the chemotherapy plus anti-PD-L1 group was augmented with anti-CD73. Withholding aPD-L1 from the 5FU+OHP plus aCD73 combination treatment was detrimental in terms of upregulating genes related to inflammation, immune-response, and interferon gamma pathway activation. Notably, ALOX15, a gene linked to macrophage function and efferocytosis, and IL-1B were affected.

**Figure 4A.**
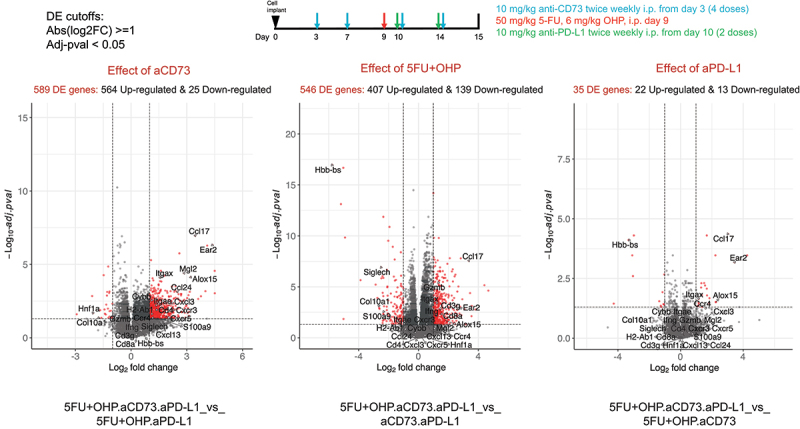
Pharmacodynamic effect of aCD73+aPD-L1+5FU+OHP with respect to CT26 transcriptome. RNAseq analysis was used to see the changes in the CT tumor transcriptome. Upper panel shows the schematic of the experimental design. Lower panel shows contribution of the individual components in the in combined aCD73, aPD-L1 and 5FU+OHP treatment vs the aCD73 + aPD-L1 group. As seen in left bottom panel, addition of aCD73 had the most profound effect leading to 589 differentially expressed (DE) genes as opposed to aPD-L1 which lead to only 35 DE genes (bottom right panel). Addition of the chemotherapeutic component to antibody doublet (aCD73 + aPD-L1) lead to 546 DE genes. DE cut-off values used were Abs(log2FC) ≥1 and Adj-pval < 0.05.

Next we checked if the gene perturbation at this scale also led to changes in the enumeration of the cell populations. For this we estimated the abundance of immune cell populations in the treated CT26 tumors, using the MCP-Counter tool^11^ as well as the imaging mass cytometry (IMC). Computational analysis using the MCP-Counter tool highlighted a prominent effect of 5FU+OHP chemotherapy, in terms of increased lymphocyte representation; in line with significantly elevated proinflammatory and chemotactic chemokine/cytokine gene expression levels following this treatment (Figure 4c). This was also corroborated by IMC results where tumors presented lower frequencies of cells expressing macrophage markers like CD68, F4/80 as well as suppressive tumor associated macrophage markers like CD163 and CD206 (Supplementary Figure S5). CT26 tumors from mice treated with monotherapies and combinations excluding the chemotherapy component were notably less infiltrated with lymphocytes. The 5FU+OHP+aCD73+aPD-L1 treatment stood out prominently for the elevated tumoral abundances of both lymphocytes (cytotoxic T-cell, NK, B-cells) and myeloid cell populations (including monocytic dendritic cells); a profile conducive to improved tumor control and increased overall survival times in cancer models.^12–15^

**Figure 4C.**
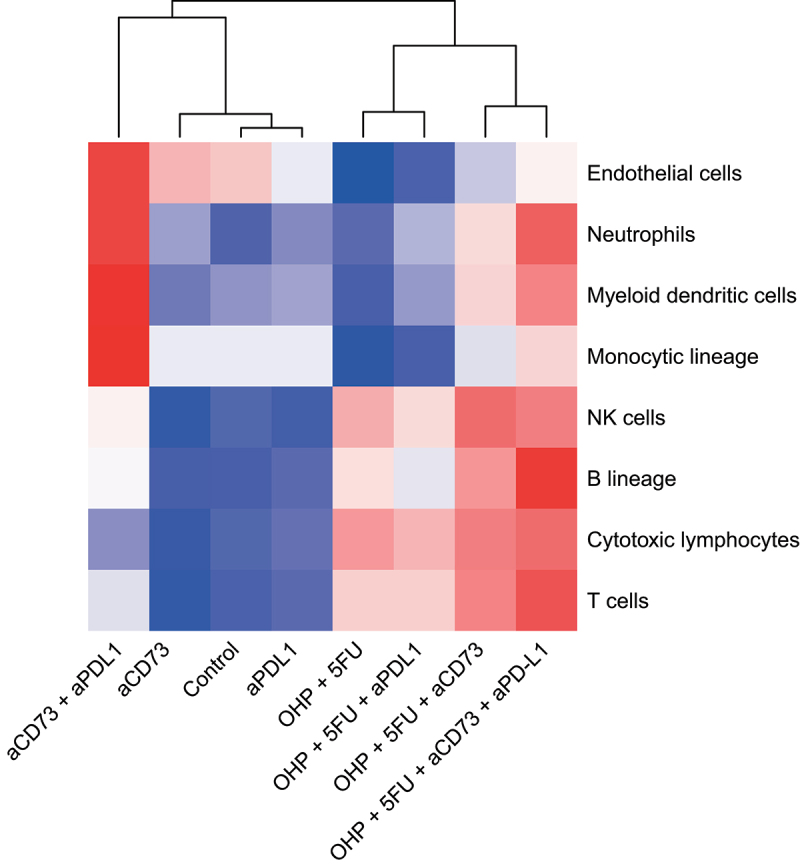
Addition of aCD73 to aPD-L1 + 5FU+OHP drove elevated tumor infiltrating lymphocytes (cytotoxic T-cells, NK, B-cells) and myeloid dendritic cells in CT26 tumors. Using the MCP counter tool the abundance of the different cell populations was estimated. As shown in the heatmap, addition of aCD73 had the most profound effect on driving the relevant immune cells like- cytotoxic T-cells, NK- cells, B-cells and myeloid dendritic cells (DC). The DC infiltration effect was solely mediated by addition of the aCD73 to the 5FU+OHP, as addition of aPD-L1 to 5FU+OHP didn’t result in increased DC infiltration in the tumors.

### Combined aCD73, aPD-L1 and docetaxel treatment leads to enhanced complete responses in CT26 syngeneic mouse model

**Figure 5.**
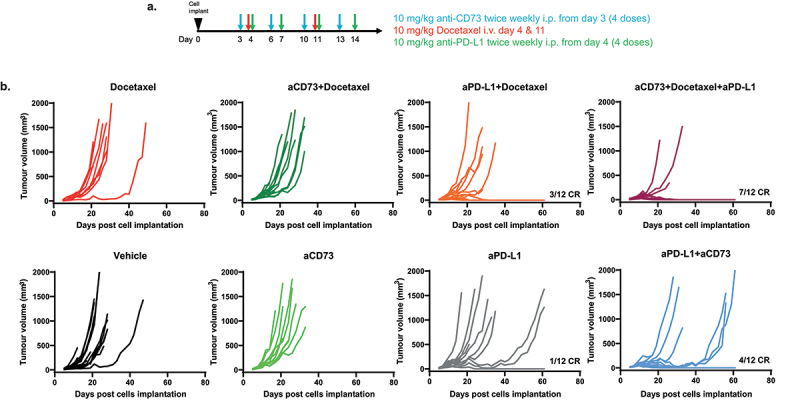
Combined aCD73, aPD-L1 and docetaxel (DTX) treatment leads to enhanced complete responses in CT26 syngeneic tumour model. (a) schematic of the experimental design. (b) BALB/c were implanted with 5e5 CT26 cells in PBS and treated as shown in schematic. Growth curves were plotted from calliper measurements done thrice weekly. Addition of aCD73 and aPD-L1 to docetaxel lead to significant (*p*=.0001 vs vehicle) increase in the number of complete responders (CR) 58% compared to 25% CR seen upon addition of aPD-L1 alone to docetaxel (*p*=.001); Kruskal Wallis test.

Importantly, the additive combinatorial effects noted with 5FU+OHP were found to extend to a second chemotherapy class; i.e. Docetaxel (DTX). We overlayed the combination approach to an already established tolerated dosing scheme for this chemotherapy (scheme in Figure 5a). In this case the combination of DTX, aPD-L1 and aCD73 resulted in significantly improved tumor growth-inhibition and in 7 out of 12 (58%) complete responses (p < .0001 vs DTX alone) compared to a maximum of 3 out of 12 (25%) complete responses in the aPD-L1 plus docetaxel combination group (p = .004, vs DTX alone) Kruskal-Wallis test, as shown in Figure 5b.

### Fractionated RTx is enhanced by combined aCD73, aPD-L1 in MC38 syngeneic mouse model

**Figure 6.**
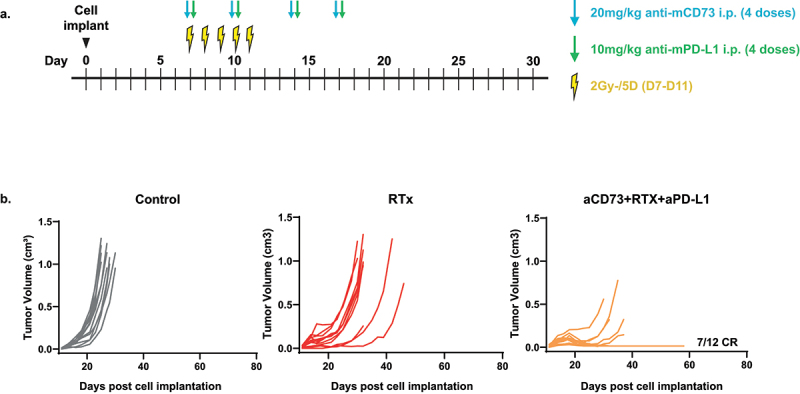
Concurrent treatment with aCD73, aPD-L1 and RTx leads to enhanced complete responses in MC38 syngeneic tumour model. (a) schematic of the experimental design. (b) C57BL6/J were implanted with 5e5 MC38 cells in PBS and treated shown in schematic. Growth curves were plotted from calliper measurements done thrice weekly. Concurrent treatment with aCD73, aPD-L1 and RTx lead to significant (*p*=.0001 vs NT) number of complete responders (CR) 58% compared to none see in RTx alone group (*p*=.5 vs NT), Kruskal Wallis test.

Previously published preclinical data highlights a role for CD73 within RTx responsiveness in cancer.^4,16,17^ We explored whether addition of aCD73+aPD-L1 treatment would also augment RTx responses in line with our chemotherapy data. We tested this using the MC38 model of colorectal cancer, already established in our hands for fractionated RTx regimens, employing a concurrent approach in this experiment, i.e., all the treatments starting on the same day (schematic Figure 6a). The data confirmed a potent effect (p < .0001 vs RTx alone, Kruskal-Wallis test) from RTx+aCD73+aPD-L1 treatment of MC38 tumor bearing mice as shown in Figure 6b.

## Discussion

A growing body of evidence supports an immunosuppressive role for extracellular adenosine within the tumor microenvironment; with adenosine related gene signatures linked to poor outcome and reduced response to T-cell checkpoint inhibiting drugs in several indications.^1,3,18^ Molecules targeting extracellular nodes within the adenosine creation pathway have gained traction in the clinical development space, with Oleclumab now in Ph3 of clinical development in combination with Durvalumab in stage 3 NSCLC patients previously treated with chemo-radiotherapy.^9^

Despite the widespread assumption that CD73 inhibition will combine beneficially with cytotoxic treatments that promote extracellular ATP release via cell death, there is a paucity of published preclinical data to support this. We have attempted to fill some of this knowledge gap and provide deeper mechanistic insight to assist clinical translation line-of-sight. Our preclinical data, exploring the effects of CD73 inhibition in concert with chemotherapy and PD-L1 inhibition, highlight additivity and novel biological effects mediated by inclusion of CD73 blockade. To this end, we found a combination comprising aCD73+aPD-L1 + 5FU+OHP afforded enhanced efficacy in two mouse models of cancer (colorectal and sarcoma). As is the case with immunotherapies in the clinical setting, there was a subset of non-responding mice whose tumors grew regardless of the treatment given. Such findings are consistent with the expected tumor and biological heterogeneity, even in a syngeneic model. The combination therapy activity observed was contingent on CD8 T-cells, as judged by the effect of a CD8 depleting antibody in the MCA205 model. These data infer a critical contribution from the cell mediated arm of the murine immune system in the anti-tumor effect engendered. Consistent with this, RNAseq analysis confirmed aCD73+aPD-L1 + 5FU+OHP drove elevated tumoral abundances of cytotoxic lymphocytes and other key immune-cells, such as myeloid dendritic cells and B cells. We also explored substituting OHP + 5FU with a taxane backbone, i.e. DTX, and confirmed a similarly enhanced efficacy profile was attained in the CT26 model. These preliminary preclinical data support complementarity of Oleclumab and aPD-L1 antibodies, with both Platin- and Taxane-based chemotherapy backbones. Our data also highlight additivity with RTx in the MC38 model; extending the findings of Wennerberg *et al* ^4^ to colorectal models and augmenting the interventional strategies with PD-1/PD-L1 axis inhibition using clone 80.

Mechanistically we confirmed that the aCD73 antibody rapidly lowered CD73 expression within CT26 tumors and modulated extracellular adenosine levels in manner consistent with its proposed mechanism of action. We also highlighted interesting pharmacodynamic changes with other metabolic biomarkers including inosine and xanthine; although we did not detect any elevation in ATP following chemotherapy treatment. Imaging mass cytometry of the same samples did, however, pick up changes in CAF and TAM markers, that may reflect direct or downstream effects of aCD73 treatment. Such observations align with other publications linking CD73 inhibition to tumoral macrophage and fibroblast representation.^19,20^

Monotherapy 5FU+OHP treatment retarded tumor growth in a proportion of drug recipients bearing CT26/MCA205 tumors. A notable finding from our current studies was the breadth of immune-pathway gene modulation following 5FU+OHP treatment of CT26 tumors. These included: Type 1 and 2 interferons, gene signatures associated with cytotoxic lymphocytes and effector molecules and their related receptors. Such observations align with many of the known immunomodulatory effects of these chemotherapies within *in vitro* model systems^21^ and now extend the findings concerning effects *in vivo*.^22^ Our data have identified 5FU+OHP drives type I interferon pathways, which are known to exert wide ranging effects on immune and cancer cells within the tumor microenvironment.^23^ Specific genes modulated by monotherapy 5FU+OHP treatment included IFN-γ, CCL3, CCL8, LAG3 and Granzyme B (upregulated) with downregulation of CXCL2, IL-1B, CD103 and XCR1. 5FU+OHP treatment elevated ARORA2 gene expression, although ARORA2 upregulation was counteracted when either aPD-L1 or aCD73 Mabs were combined with 5FU+OHP treatment. Gene signatures associated with proinflammation, STAT5 pathway activation and chemotaxis were only significantly upregulated once a CD73 blockade was applied with the OHP + 5FU treatment (Supplementary Table S2 and Figure S6). GDF-15 is a pleiotropic cytokine of emerging interest in cancer,^24^ and we saw that GDF15 transcript levels in CT26 tumors were markedly elevated by 5FU+OHP containing treatments; mirroring findings from other mouse models and human cancer patients on platinum therapy.^25^

In contrast to the tumor RNAseq data obtained from mice treated with either of the IO agents alone (e.g., as monotherapies), the combination of aCD73 and aPD-L1 resulted in 1236 differentially expressed genes; highlighting broader transcriptomic changes associated with antibody mediated targeting of multiple inhibitory checkpoints within the tumor microenvironment. The IO ‘doublet’ was noted for its activation of pathways and gene families linked to inflammation, myeloid leukocyte migration, cell chemotaxis, cytokine-cytokine receptor interactions (including TNF), chemokine signaling pathways and complement and coagulation cascades. The pathways and processes influenced by the IO combination (aCD73+aPD-L1) were mostly distinct from those influenced by 5FU+OHP treatment, with potential for complementarity if overlayed within a combinatorial therapeutic paradigm. Notably, the ‘doublet’ IO combination (aCD73+aPD-L1) afforded less efficient activation of type 1 interferon pathway genes; known to correlate with favorable disease outcomes in patients with many forms of cancer and mediate a range of beneficial immunomodulatory effects within the tumor microenvironment.^23^ Ultimately it would be informative to explore whether there is a detrimental effect of neutralizing specific cytokines linked to these immunomodulatory pathways; through use of blocking antibodies or knock-out mice.

Despite the detected transcriptomic changes, ‘purist’ small/large molecule approaches (5FU+OHP chemotherapy or IO combinations) failed to attain the same level of efficacy afforded by combined use of 5FU+OHP with aCD73 and aPD-L1. It was therefore informative to drill down into the specific differences in the transcriptomes that could account for this, as these genes and these signatures might be useful beyond the context of adenosine pathway modulation. Strikingly, addition of aCD73 to 5FU+OHP + aPD-L1 significantly upregulated CXCR3 in the CT26 tumor microenvironment. CXCR3 is the cognate receptor on activated T-cells for the IFN induced chemokines CXCL9–11; its upregulation being coincident with elevated abundances of T-cells and increased expression of interferon activated chemokines in the CT26 tumors of mice that received 5FU+OHP, or combinations containing that chemotherapy. We also note with interest that recent publications highlight the importance of tumoral chemotaxis of CXCR3 bearing T-cells for preclinical and clinical responsiveness to T-cell checkpoint inhibitors.^26–28^ Elevated expression of PDCD1 (PD-1) infer tumoral T-cells in those CT26 tumor bearing mice are likely activated and/or exhausted and strongly support the inclusion of aPD-L1 to counteract adaptive immune-resistance to anti-tumor effects. Remarkably, the aPD-L1 and IO containing combinations markedly upregulated 15-lipoxygenases (15-LOXs), which have been implicated in various macrophage functions including efferocytosis and ferroptosis. This is interesting in that Snodgrass *et al*.^29^ identified a novel role for ALOX15 in CCL17 production in human macrophages; important to note considering the obvious increase in CCL17 expression in the most protective therapeutic formats.

The regulation of genes linked to dendritic cell biology and antigen presentation (MHC II, ITGAE, ITGAX, DCSTAMP, TARM1, CD301) is particularly interesting and consistent with the work of others exploring CD73 inhibition in the context of RTx^4^ and adenosine pathway blockade in general.^3,4^ Wennerberg *et al*.^4^ already highlighted the critical role of RTx induced type 1 interferons for reprofiling the tumor microenvironment, particularly with respect to cDC1’s. The same group highlighted the complementarity for CD73 blockade with RTx, with a critical role for aCD73 treatment in tumors with suboptimal induction of radiation elicited type 1 interferons. Our data seem broadly in line with their findings. Consistent with this, genes for MHCII molecules and the macrophage galactose C-type lectin (MGL/CD301), expressed on DCs, were upregulated within the CT26 TME. CD301 is thought to participate in the recognition of molecules from both altered self and pathogens due to its monosaccharide specificity for Gal and N-acetylgalactosamine.^30^ T cell-interacting, activating receptor on myeloid cells-1 (TARM1; gene symbol *Tarm1*) is a recently identified LILR family member encoded within the leukocyte receptor complex. TARM1 is expressed by and is required for the activation of DCs; *Tarm1* was highly expressed in inflammatory-type (I-A/I-E^+^Ly6C^+^CD11b^+^CD11c^+^) DCs in draining LNs (dLNs) after induction of CIA in *Tarm1*^±^ mice.^31^ Another novel finding was the ability of the aCD73 containing combinations to uniquely drive high levels of Ear2 expression. Ear2 is an RNase and also forms part of a 14 gene signature expressed by non-classical monocytes.^32^ Emerging evidence points to an immunomodulatory role for extracellular RNase molecules, thereby acting as alarmins.^33^ Similarly, tumoral RNase2a expression was significantly increased by the combination therapy. An effect on NOX2 (gene CYBB) was also noted, and potentially noteworthy, considering its fundamental role in conferring macrophages with the ability to respond to extracellular ATP stimulation with robust changes in cellular oxidation^34^ and its role in modulating ATM kinase activation in macrophages and effectiveness to RTx therapy.^35^ Macrophage lectin-like oxidized LDL receptor-1 (LOX-1/OLR1) was also upregulated in CT26 tumors from mice treated with 5FU+OHP+aCD73+aPD-L1. This receptor is known to sense heat-shock proteins and is markedly upregulated by TLR agonists and other proinflammatory stimuli.

The effect on B cell representation within the CT26 tumor is also comment worthy (Figure 4a,c). B-cell related genes, for example JChain, CXCR5 and CXCL13 were clearly modulated when aCD73 was included in the 5FU+OHP+aPD-L1 combination (Supplementary Fig S7). Although the influence of B-cell biology is less well understood than CD8s in cancer, there is growing interest in the role of B cells in human tumor microenvironments.^36–38^ The murine B cell-related data are also concordant with recent publications concerning direct effects, of CD73 inhibiting antibodies, on human B-cells.^39,40^ These mechanistic data align nicely to the enhanced survival rates and tumor growth control profiles in animals dosed with 5FU+OHP+aCD73+aPD-L1.

## Material and methods

### Animal studies

In vivo studies were performed using 8–10 weeks old BALB/cAnNCrl mice (Charles River UK) or C57BL/6. Mice were housed in AstraZeneca vivarium, accredited to Association for Animal Accreditation of Laboratory Animal Care, with free access to food and water *ad libitum* and were cared for daily by trained personnel. Mice were acclimatized to the vivarium conditions for a week and handled according to the Home Office Animals Scientific Procedures Act, 1986, UK. All animal work was carried out under a Home Office approved project license (PPL P49077891 and PP3208003) as well as according to the institutional guidelines. BALB/c mice were subcutaneously implanted with 0.5e6 CT26 (colorectal) tumor cells. C57BL/6 mice were subcutaneously implanted with 0.5e6 MCA205 (fibrosarcoma) tumor cells in 50% QuantaBlu™ (Corning, Cat. No. 356231) in PBS or with 0.5e6 MC38 cells in PBS. Tumors were measured with callipers three times a week, starting from day-5. Tumor bearing mice were treated either with combinations of oxaliplatin (Hospira, Clinical grade, 5 mg/mL) at 6 mg/Kg dose level and 5-Fluorouracil (Hospira, Clinical grade, 50 mg/mL) at 50 mg/Kg dose level or docetaxel (Accord, Clinical grade, 20 mg/mL) dosed at 10 mg/Kg along with murine surrogate monoclonal antibodies for Oleclumab (clone 10.3, an anti-CD73 with murine IgG1 Fc sequence, 10 mg/Kg or 20 mg/Kg) and Durvalumab (clone 80, a chimeric rat anti-mouse PD-L1 antibody with IgG1 Fc sequence, 10 mg/Kg). The murine surrogate for Oleclumab/MEDI9447 contains identical variable domains to Oleclumab. There is high amino acid sequence homology (86%) for human and murine CD73 and Oleclumab binds to human and murine CD73 with similar affinity (KD: 154 × 10–12 and 113 × 10–12 M) as determined by flow cytometry and Surface Plasmon Resonance. Using ELISA-based assays we showed the binding ability of oleclumab and murine surrogate aCD73 clone 10.3 mIgG1 to both human and mouse CD73. Both antibodies (human and mouse) bind to the respective CD73 proteins with equal affinities (Supplementary Fig. S8). The dose and schedule of chemotherapy employed were based on previously published information.^22,41^ To define contribution of components, comparator groups received monotherapies and other iterations of the combination. Groups of mice were removed for pharmacodynamic analysis MSI, and transcriptomics) as well as immunohistochemical (IHC) analysis at appropriate timepoints. For the RTx experiments, tumors were treated with five doses of 2 Gy fraction given on each consecutive day using X-ray source Self-Contained Cabinet Irradiator (Xstrahl CIX3). The tumors were enrolled once they were between 70 mm^3^ and 120 mm.^3^

### In vitro assays

Cells (HCT-116, HT-29, CT-26 and MCA-205) are seeded in 96-well culture plates at 1e^4^ cells/well in 40 µl of complete culture media and cultured in a 37°C/5%CO_2_ incubator for about 4 hours to allow cell adherence. 10 µl/well of Oleclumab (5 nM) at 5× final concentration is added to the cells and cultured in a 37°C/5%CO_2_ incubator overnight to allow Oleclumab pre-treatment. 50 µl/well of serially diluted chemotherapeutic drugs (5-Fluorouracil, Oxaliplatin, Docetaxel) at 2× final concentrations are added to the cells, in accordance to the treatment groups and cultured in a 37°C/5%CO_2_ incubator for 72 h. Cell viability is measured using CellTiter-Glo® Luminescent assay (Promega, Cat. No. G7573).

### RNA preparation for bulk sequencing

Tumour tissue was flash-frozen in liquid nitrogen and stored at − 80֯C at the time of collection in the animal unit and was sent to Novogene on dry ice. Tissue processing and RNA extraction was carried out by Novogene Co. by using the QIAGEN RNeasy Plus Universal kit (Qiagen, Cat. No. 73404), according to manufacturer’s protocol. Briefly, tissue samples were homogenized in QIAzol Lysis Reagent. The homogenate was then separated into aqueous and organic phases by centrifugation after addition of gDNA eliminator solution and chloroform. The upper, aqueous phase containing RNA was collected, and RNA purified using RNeasy spin columns. RNA quality check (QC) was performed by 1% Agarose gel electrophoresis, amount and purity were measured by Nanodrop, and RNA Integrating Number (RIN) was obtained by Bioanalyzer Agilent2100.

NEBNext® Ultra RNA library prep kit (Cat. No. E7530L) was used for library preparation for sequencing. Samples were sequenced using Illumina PE150 (50 M reads/sample) bulk RNA-seq and downstream bioinformatic analysis was performed.

### Analysis of RNASeq data

Reads were mapped to Mus musculus genome (mm10) genome using Star.^42^ Uniquely mapped reads were counted using htseq-count.^43^ We used DESeq2^44^ to normalize counts with size factors and identify differentially expressed genes across conditions with a threshold of abs(log_2_FC)>1 and adjusted *p* value < 0.05.

Gene set enrichment analysis was performed using R package ‘fGSEA’^45^ using hallmark gene sets from mouse MSigDB.^46^ Enrichment p-values were calculated as described in,^45^ and p-values were adjusted using Benjamini-Hochberg method. Gene ontology Biological Process enrichments were identified using R package topGO^47^ with adj-pval <0.05. KEGG pathway enrichment analysis was conducted using R package clusterProfiler.^48^ MCPCounter tool^11^ was used to estimate immune cell abundances for each sample and condition using immune gene signatures within MCPCounter.

### Tissue preparation for mass spectrometry imaging (MSI)

Tumors were snap frozen in liquid nitrogen immediately after resection and the frozen tissues were embedded in a HPMC/PVP hydrogel as previously described.^49^ Sectioning was performed on a CM3050 S cryostat (Leica Biosystems, Nussloch, Germany) at a section thickness of 10 μm and the tissue sections were immediately thaw mounted and dried under a stream of nitrogen and sealed in vacuum pouches to preserve the metabolic integrity of the sections. Tissue sections for DESI-MSI and IMC were thaw-mounted onto Superfrost microscope slides (VWR, Cat. No. 630–2863), whilst sections prepared for MALDI-MSI were thaw mounted onto conductive indium tin oxide (ITO) coated slides (Bruker Daltonik, Cat. No. 8237001). Polyvinylpyrrolidone (PVP) and (Hydroxypropyl)methylcellulose (HPMC) (were purchased from Merck (Cat. No.PVP360; H8384). Methanol (Cat. No. 15624680), iso-pentane (Cat. No. 15692830) and isopropyl alcohol (Cat. No. 10674732) were obtained from Fisher Scientific.

### Mass spectrometry imaging (MSI)

DESI-MSI analysis was performed on a Q-Exactive mass spectrometer (Thermo Scientific, Bremen, Germany) equipped with an automated 2D-DESI ion source (Prosolia Inc., Indianapolis, IN, USA) operated in negative ion mode, covering the applicable mass range up to m/z of 1000, with a nominal mass resolution of 70,000. The injection time was fixed to 150 ms resulting in a scan rate of 3.8 pixel/s. The spatial resolution was set to 70 µm. A home-built Swagelok DESI sprayer was operated with a mixture of 95% methanol, 5% water delivered with a flow rate of 272 1.5 μL/min and nebulized with nitrogen at a backpressure of 6 bar. The resulting .raw files were converted into .mzML files using ProteoWizard msConvert (version 3.0.4043) and 274 subsequently compiled to an.imzML file (imzML converter version 1.3).^50^ All subsequent data processing was performed in SCiLS Lab (version 2021b, Bruker Daltonik, Bremen, Germany).

MALDI-MSI analysis was performed on a RapifleX Tissuetyper instrument (Bruker Daltonik, Bremen, Germany) operated in negative detection mode. 9-Aminoacridine (9-AA) prepared in 80:20 methanol:water was used as a MALDI matrix and spray deposited using an automated spray system (M3-Sprayer, HTX technologies, Chapel Hill, NC, USA). MALDI experiments were performed with a spatial resolution of 50 μm. A total of 400 laser shots were summed up per pixel to give the final spectra. For all experiments, the laser was operated with a repetition rate of 10 kHz. All raw data was directly uploaded and processed in SCiLS lab (Version 2021b) software packages. All DESI and MALDI data and images were normalized to the total ion current (TIC) to compensate for signal variation across the course of the experiments. The data analysis performed in the SCiLS lab software packages included classification of the datasets based on manual annotation of the MSI data guided by the histology to identify “tumor”, and “necrosis” whilst removing the background as applicable. The tissue classification was performed using partial least squares-discriminant analysis (PLS-DA). The “necrotic margin” was delineated and differentiated from the “viable tumor” by subjecting the “tumor” tissue cluster to a pixel-wise principal component analysis (PCA). The loadings of principal components (PCs) highlighting the tissue compartment adjacent to necrotic areas were extracted and used to create a peak list for unsupervised segmentation based on a bisecting K-means classifier. All data shown was extracted from the “viable tumor” cluster.

### Imaging mass cytometry (IMC)

Imaging mass cytometry was performed on a slide which had been analyzed by DESI-MSI. Antibodies used for IMC staining are shown in supplementary Table S1. Antibodies were either purchased pre-conjugated with a heavy-metal tag and untagged antibodies were conjugated in house, using Fluidigm Maxpar Antibody Labelling Kit, according to manufacturer’s instructions. The slides were fixed with 4% paraformaldehyde in phosphate-buffered saline (PBS) for 10 minutes. The slides were washed 3 × 5 minutes in PBS, permeabilized for 5 minutes with 1:1000 dilution of Triton X-100 in Casein Solution, washed 3 × 5 minutes in PBS, and blocked for 30 minutes with Casein Solution. Antibodies were diluted to an appropriate concentration and the slides incubated overnight with the antibody solution at 4°C. The slides were washed 3 × 5 minutes in PBS and nuclei were stained with DNA Intercalator-iridium at a dilution of 1:400 in PBS for 30 minutes. The slides were washed 3 × 5 minutes in PBS, 30 seconds in deionized water, then dried for storage at room temperature until analysis. A region for IMC analysis was selected using consecutive H&E stained sections and the MSI results. Areas of approximately 1.5 × 1.5 to 2.0 × 2.0 mm were selected for analysis to include necrotic, necrotic margin and viable tumor regions. IMC analysis was performed using a Hyperion instrument (Fluidigm Corporation, San Francisco, CA, USA) with an ablation energy of 4 db an ablation frequency of 100 Hz.

IMC data were analyzed using Halo v.3.3.2541.366 (Indica Laboratories, Albuquerque, NM, USA). Tissue regions were classified into viable tumor, necrosis, necrotic margin and off-tissue using Random Forest. Analysis was performed using Highplex FL v4.0.4. Cell segmentation and thresholds were optimized manually. Nuclear segmentation was performed using 193Ir DNA intercalator channel. IMC images were adjusted and extracted using the build-in figure maker tool.

### Immunohistochemistry

A portion of each tumor was immersion-fixed in 10% neutral-buffered formalin, then processed to paraffin using routine methods. Tissues were sectioned at 4 µm thickness and immunohistochemically stained using a rabbit monoclonal antibody to CD73 (D7F9A, Cell Signaling Technology) at a 0.5 ug/ml dilution, on an automated Leica Bond-RX immunostainer, using DAB as a chromogen and hematoxylin as background stain. This antibody was previously shown not to have binding inhibition in tissues previously treated with Oleclumab (unpublished observations).

Resulting slides were digitally scanned at 20× magnification using an Aperio scanner (Leica Biosystems). For analysis, measurement of positive CD73 area and of hematoxylin-positive area were performed using Halo image analysis software (Indica Labs). Briefly, the tumors areas were manually annotated, and the positive DAB and hematoxylin areas were measured in the annotated area using the Halo area quantification algorithm, adapted to the staining characteristics of the tissues. Percent of CD73-positive area and hematoxylin-positive areas within the total tumor area were obtained. The final data output used was the ratio of CD73-to-hematoxylin areas, hematoxylin area being considered a representative indicator of cellularity (as it mainly stains nuclei), allowing normalization of CD73-positive area to the overall cellularity of the tumors.

### Protein binding assays

The binding ability of oleclumab and aCD73 mIgG1 clone 10.3 to human and murine CD73 was tested in an ELISA. A 96 half well plate (Corning, 3690) was coated with streptavidin (Invitrogen 434,301) at 2ug/ml overnight with no shaking, washed, and then blocked for one hour using 1% BSA (Sigma, A6003) in PBS. The plate was washed, and 0.5ug/ml biotinylated human (Amsbio, AMS.CD3-H82E3) or murine CD73 (Amsbio, AMS.79416–1) was incubated for one hour. Twelve point serial dilutions of oleclumab and aCD73 mIgG1 clone 10.3 (In house, 864–0.014632 ng/ml) were added and incubated for two hours, before incubation of 1:2500 HRP labeled anti mouse Fc antibody (Sigma, A2554), or 1:5000 HRP labeled anti-human Fc antibody (Sigma, A0170) for one hour. QuantaBlu™ Fluorogenic Peroxidase Substrate Kit (Thermo Fisher Scientific 15,169) was used according to kit instructions to detect, and fluorescence was measured using an Envision (Perkin-Elmer, Ex@315nm_EM@402 nm). Three times washes with PBS-tween occurred between steps and incubations occurred at room temperature with 450 rpm shaking, unless otherwise stated.

### Statistical analysis

All in vivo data were collated in the Excel spreadsheets and transferred to GraphPad Prism 9.00 (GraphPad Software Inc.) for graphical representation and statistical analysis. After outlier identification and Shapiro – Wilk test for normality distribution, statistical significance was determined by One-way ANOVA followed by Dunnett’s multiple comparison for comparisons of more than two groups if data were normally distributed. Kruskal-Wallis Test with Dunn’s multiple comparisons test was used if data were not normally distributed, as detailed in figure legends. Survival studies were analyzed with a log rank (Mantel-Cox) test, comparing only two survival curves at a time. *P* values were not adjusted for multiple testing.

## Conclusions

These new preclinical data highlight the potential for complementarity between CD73 inhibiting drugs, chemotherapies/radiotherapies and T-cell checkpoint inhibiting antibodies. We report interesting and translationally relevant pharmacodynamic biomarker changes that inform mechanism and can help refine clinical biomarker strategies.

## Supplementary Material

Supplemental Material

## Data Availability

Due to AstraZeneca’s vested commercial interests in this research, the supporting RNAseq data is not available.
