# Supplementary material for: Efficacy and pharmacodynamic effect of anti-CD73 and anti-PD-L1 monoclonal antibodies in combination with cytotoxic therapy: observations from mouse tumor models
Source: Cancer Biol Ther. 2024 Jan 11;25(1):2296048. doi: 10.1080/15384047.2023.2296048 (PMC10793677; doi:10.1080/15384047.2023.2296048)
Supplement: Supplemental Material [file KCBT_A_2296048_SM7420.pdf]

Supplementary Data Files

Efficacy and pharmacodynamic effect of anti-CD73 and anti-PD-L1 monoclonal antibodies in combination with cytotoxic therapy: observations from mouse tumor models.

Brajesh P. Kaistha, Gozde Kar, Andreas Dannhorn, Amanda Watkins, Grace Opoku-Ansah, Kristina Ilieva, Stefanie Mullins, Judith Anderton, Elena Galvani, Fabien Garcon, Jean-Martin Lapointe, Lee Brown, James Hair, Tim Slidel, Nadia Luheshi, Kelli Ryan, Elizabeth Hardaker, Simon Dovedi, Rakesh Kumar, Robert W. Wilkinson, Scott A. Hammond and Jim Eyles

Supplemental Fig. S1 Effect of aCD73 and aPD-L1 treatments (mono or combined) in syngeneic tumor models

50 mg/kg 5-FU, 6 mg/kg OHP, i.p. day 9
10 mg/kg anti-PD-L1 twice weekly i.p. from day 11 (5 doses)
10 mg/kg anti-CD73 twice weekly i.p. from day 3 (4 doses)

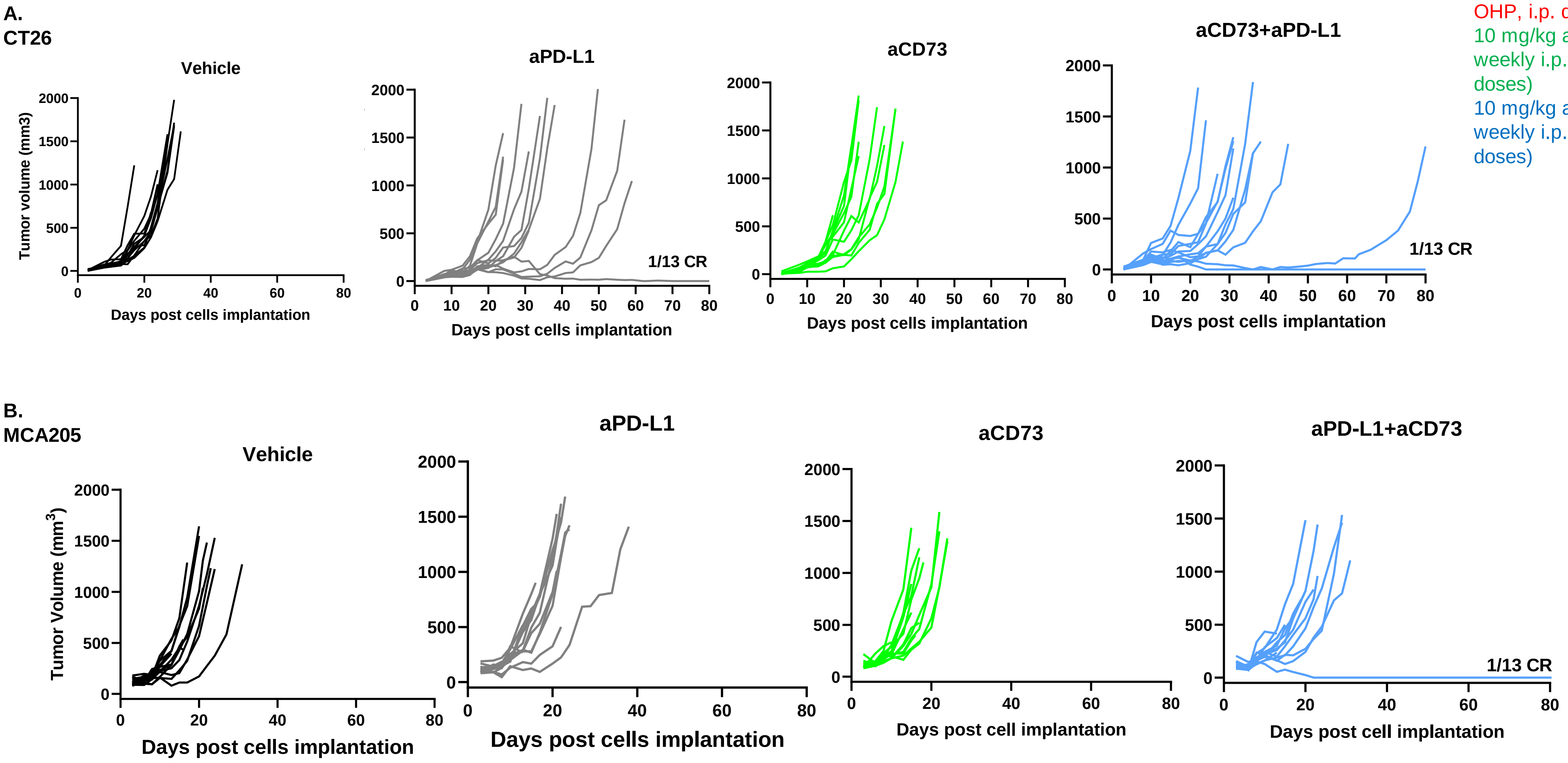

CR = complete remission

Supplemental Fig. S1 Effect of aCD73 and aPD-L1 treatments (mono or combined) in syngeneic tumor models

C. CT26

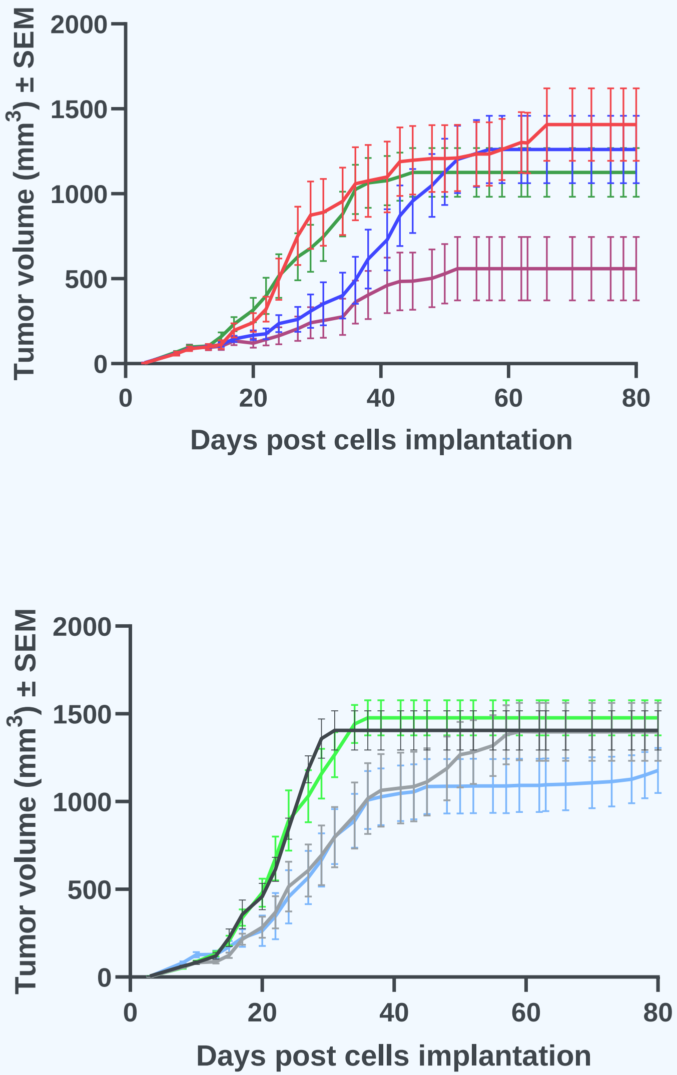

D. MCA205

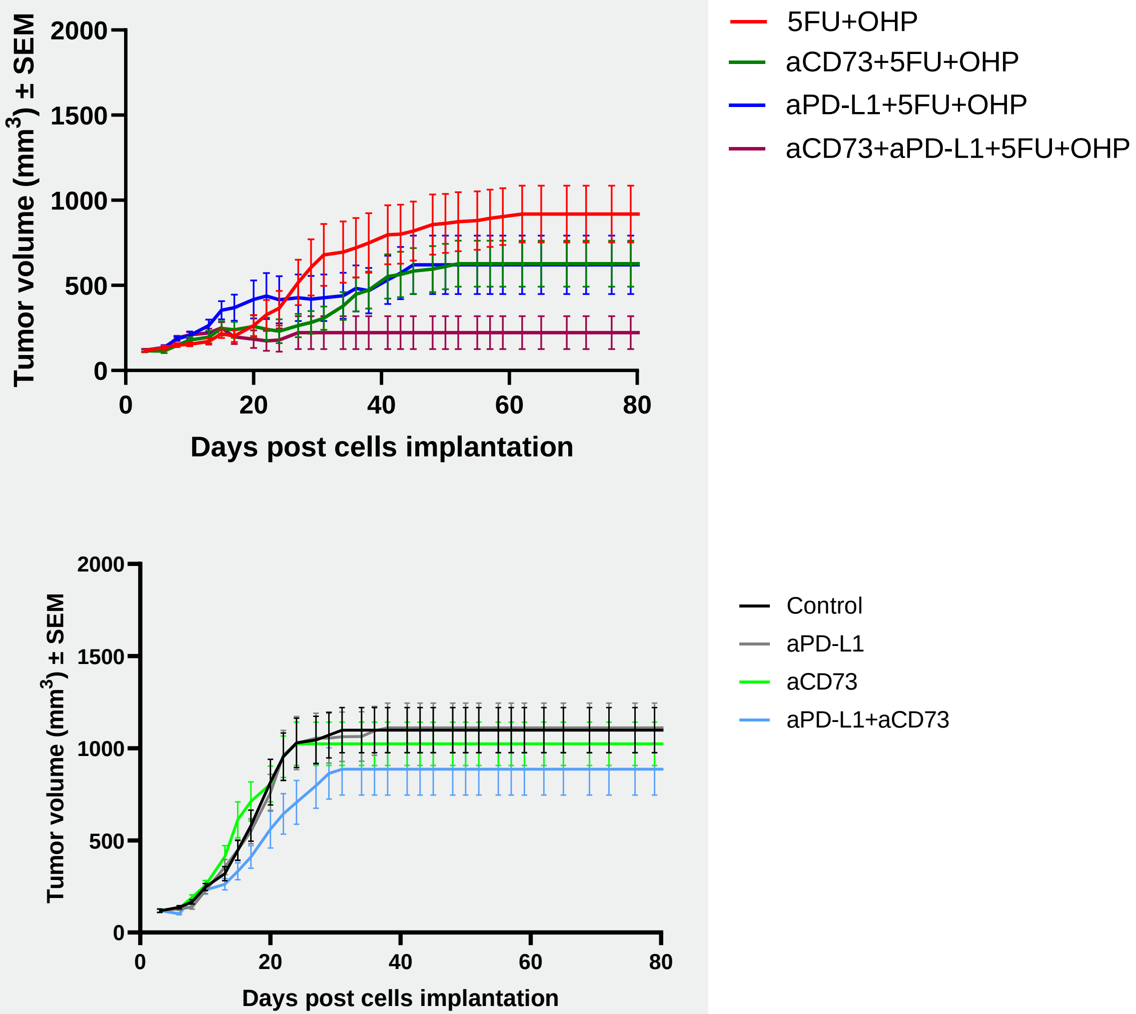

Supplemental Fig. S2. aCD73 does not enhance direct cytotoxic effect of 5FU + OHP or Docetaxel on cell lines in vitro

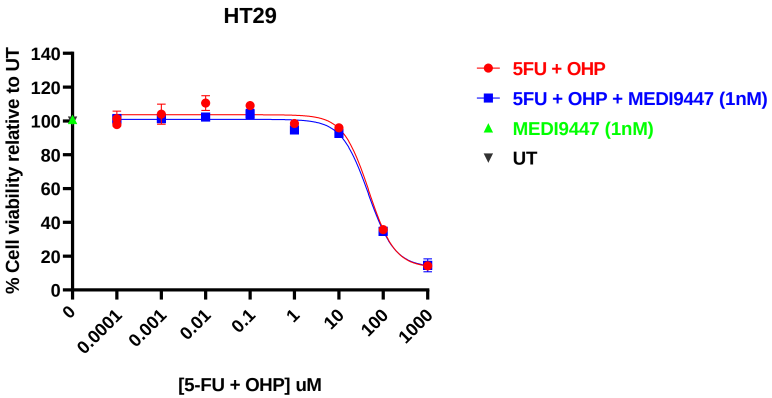

	5FU + OHP	5FU + OHP + MEDI9447 (1nM)
IC50	47.70	45.80

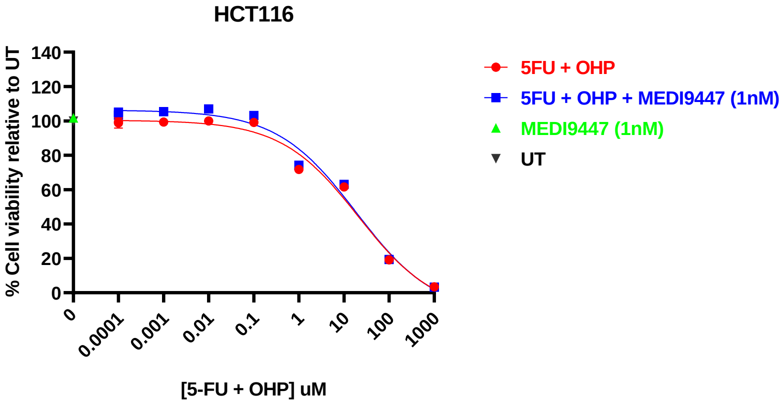

	5FU + OHP	5FU + OHP + MEDI9447 (1nM)
IC50	20.70	18.54

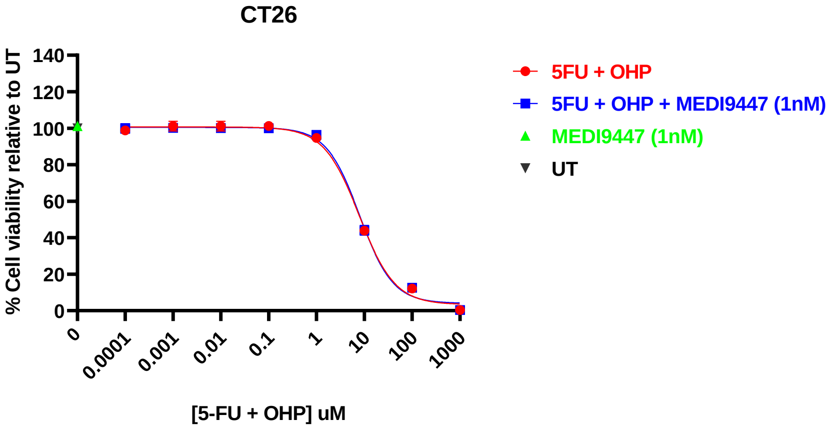

	5FU + OHP	5FU + OHP + MEDI9447 (1nM)
IC50	7.881	7.985

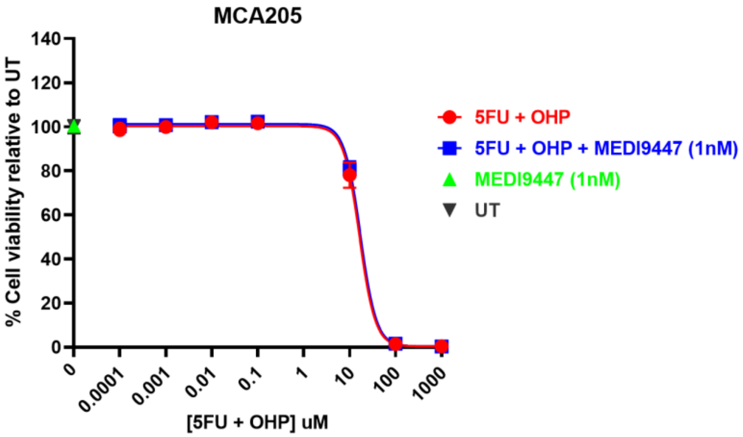

	5FU + OHP	5FU + OHP + MEDI9447 (1nM)
IC50	16.41	17.27

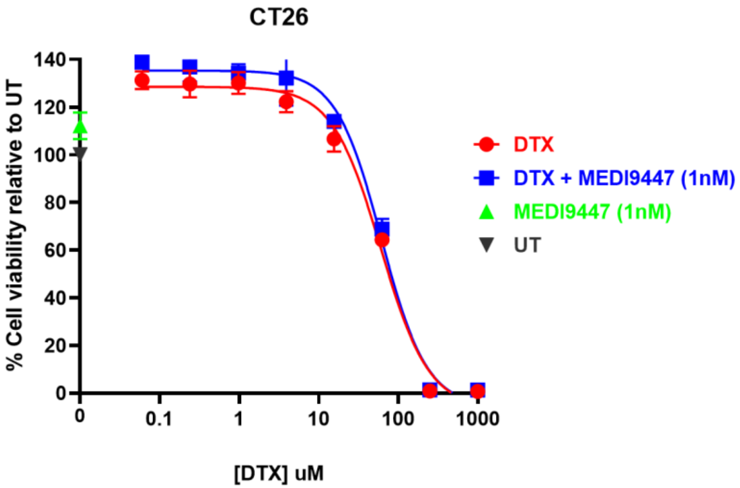

	DTX	DTX + MEDI9447 (1nM)
IC50	60.18	61.45

Supplemental Fig. S3 Flow cytometry analysis confirms increased frequencies of IFNg secreting CD8 and NK cells in aCD73 + aPD-L1 + 5FU+OHP group

A.

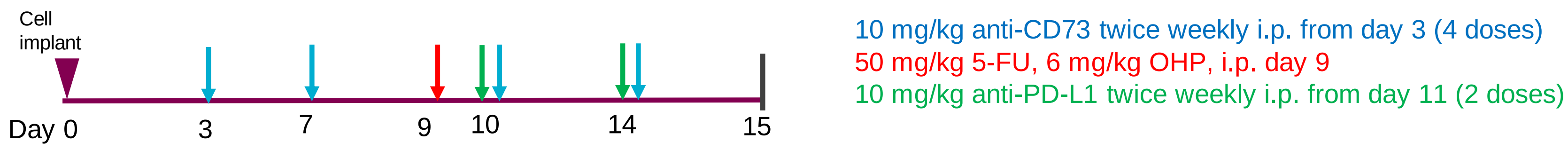

B.

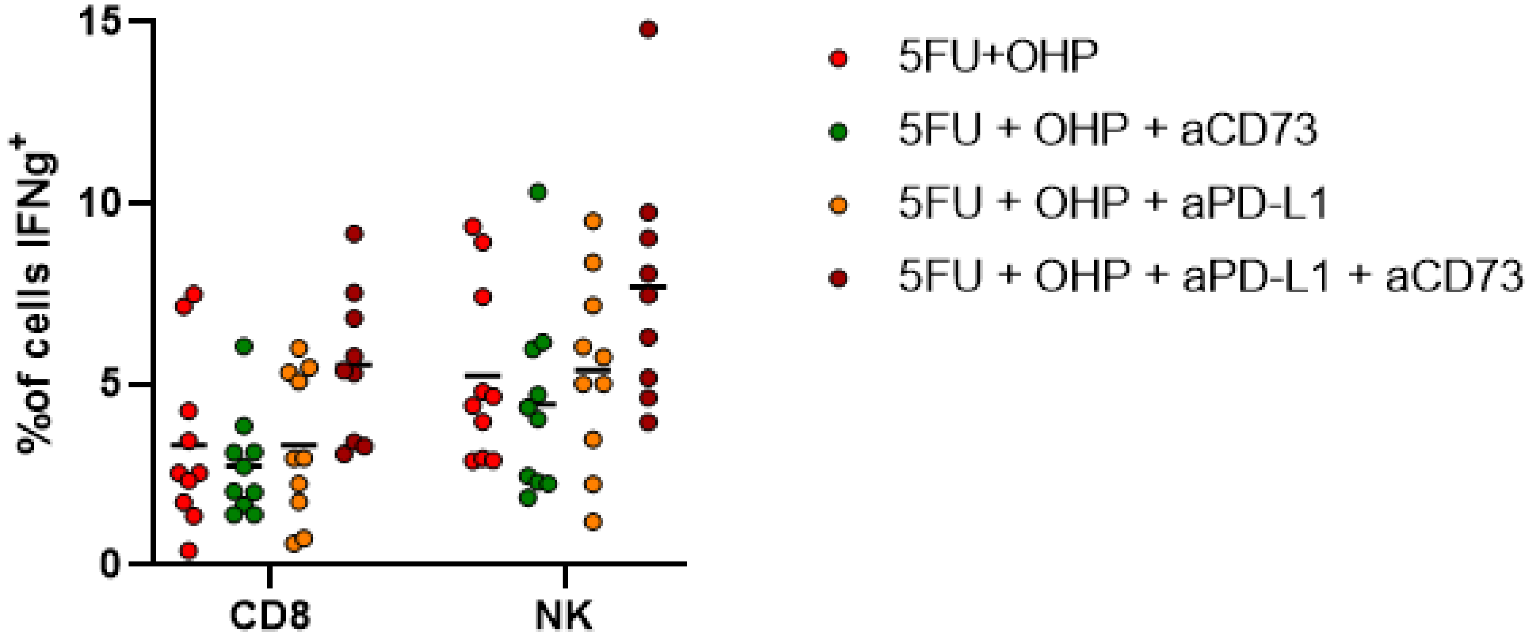

Supplemental Fig. S4 MSI analysis confirms adenosine pathway modulation by addition of aCD73 to 5FU+OHP

A.

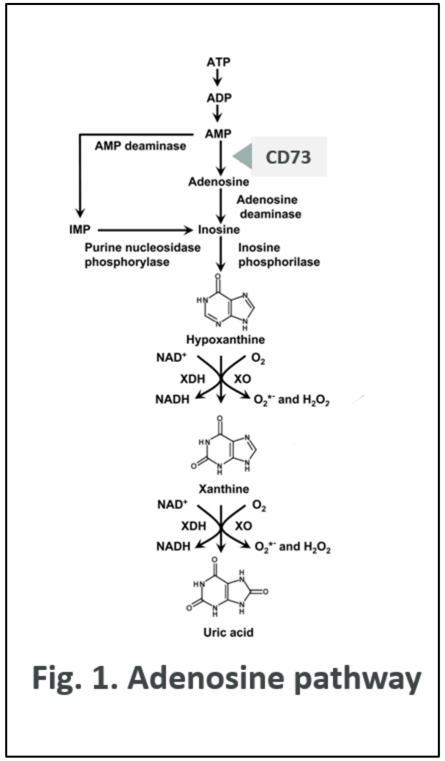

B.

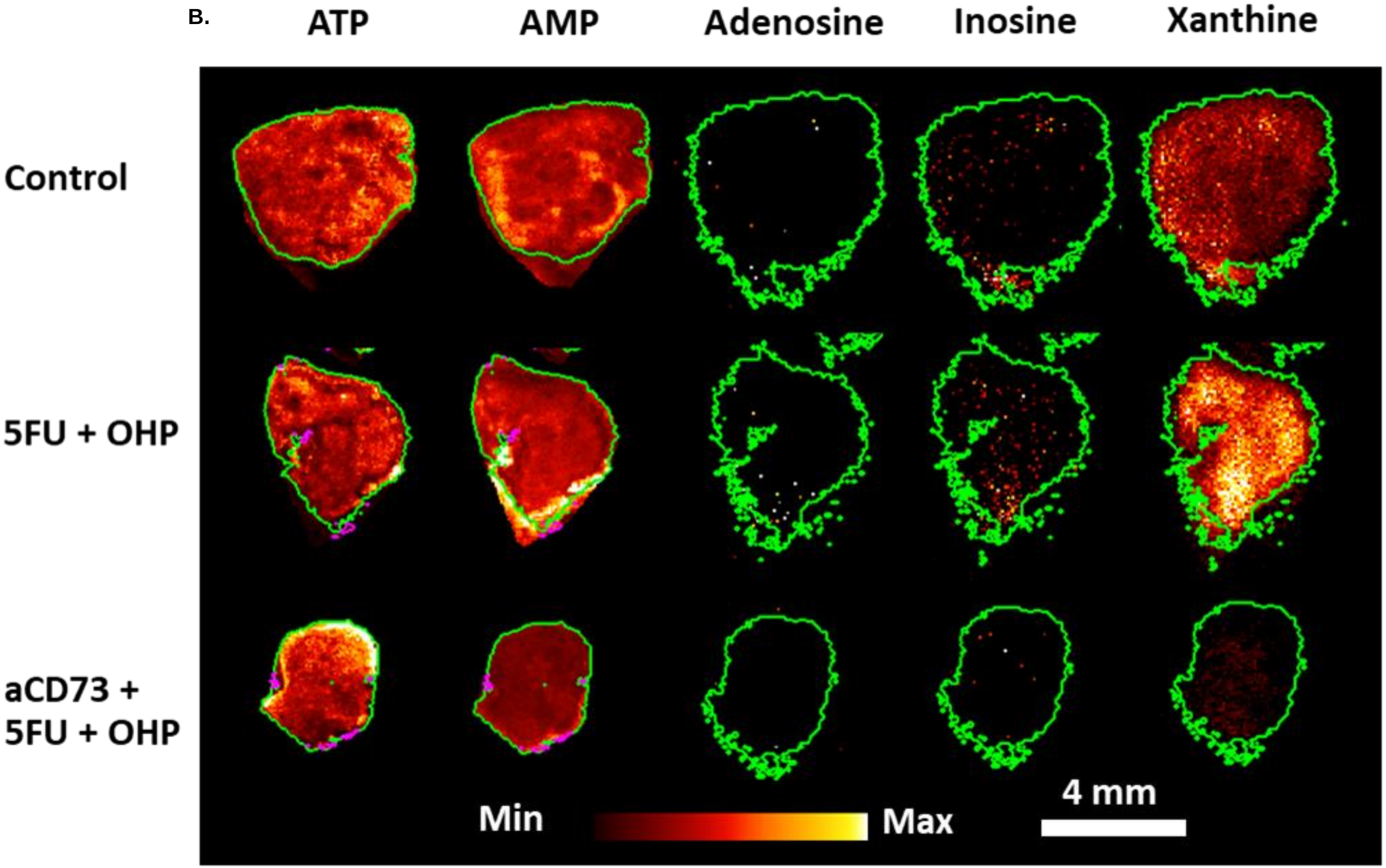

Supplemental Fig. S5 IMC highlights PD effects aCD73

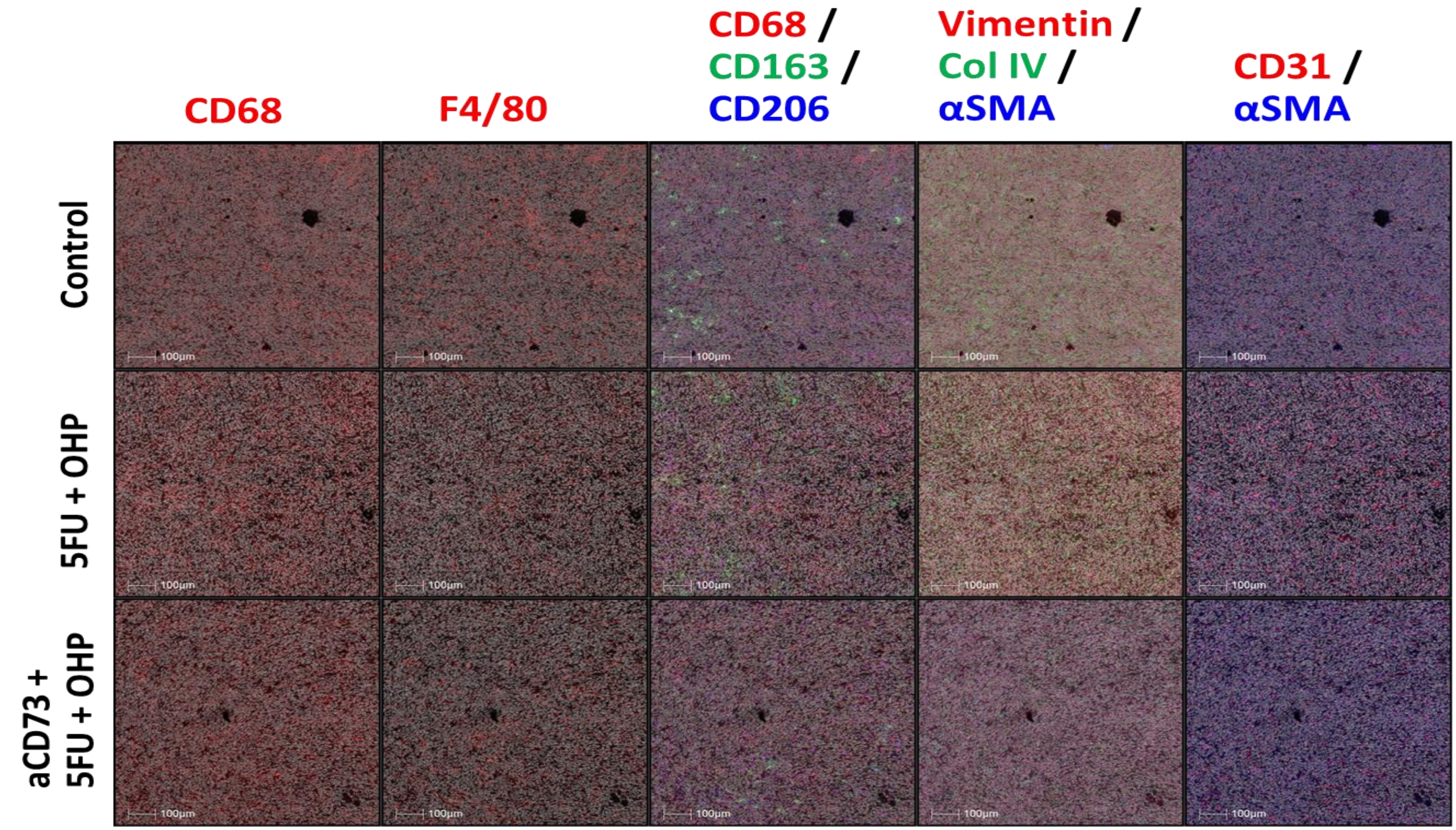

Supplemental Figure S6. Significant effects of treatments on top ranked gene signatures from MSigDB

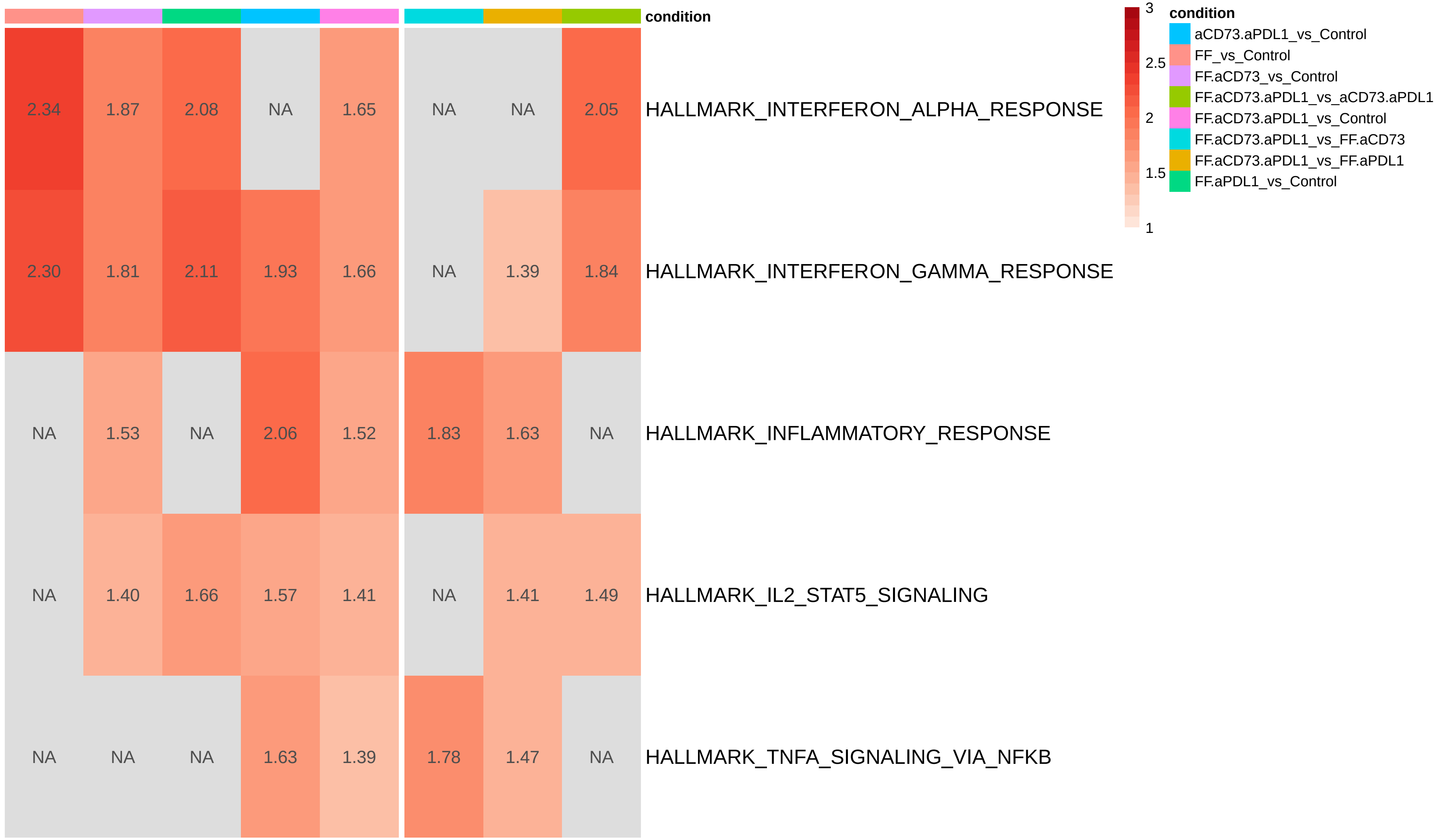

5FU+OHP drives type 1 & 2 IFN response. Adding anti-CD73 to 5FU+OHP drives up inflammation and STAT5 pathway activation (IL-2). IO doublet drives TNF-a signaling, but not IFN-1s. 5FU+OHP+aCD73+aPD-L1 drives all of the above.

Supplemental Figure S7. Combination treatments modulate many immune-related genes

Unbiased – Top 50 genes

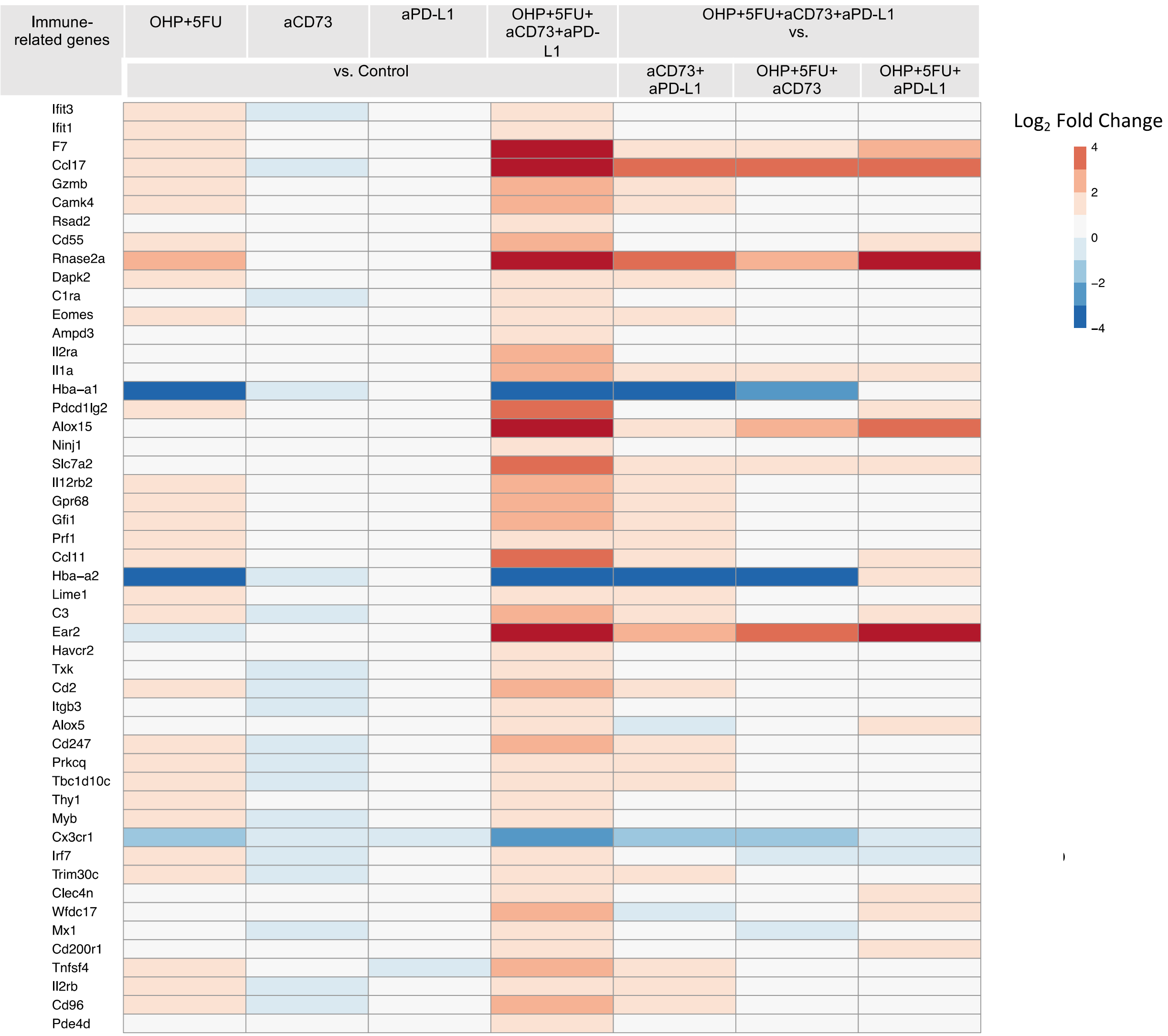

Selected – genes of interest

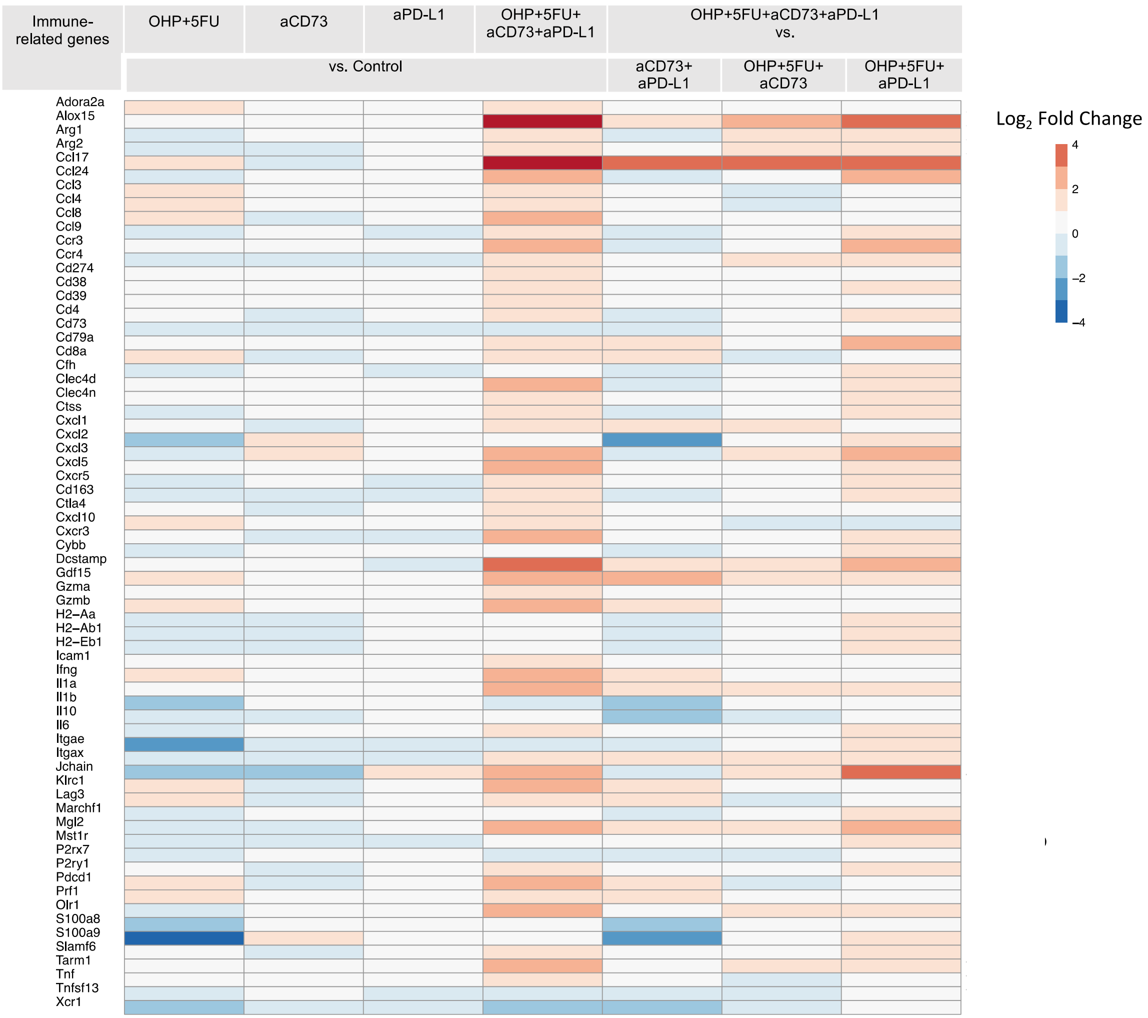

Supplemental Figure S8. Binding of Oleclumab vs aCD73 clone 10.3 mIgG1 to human CD73

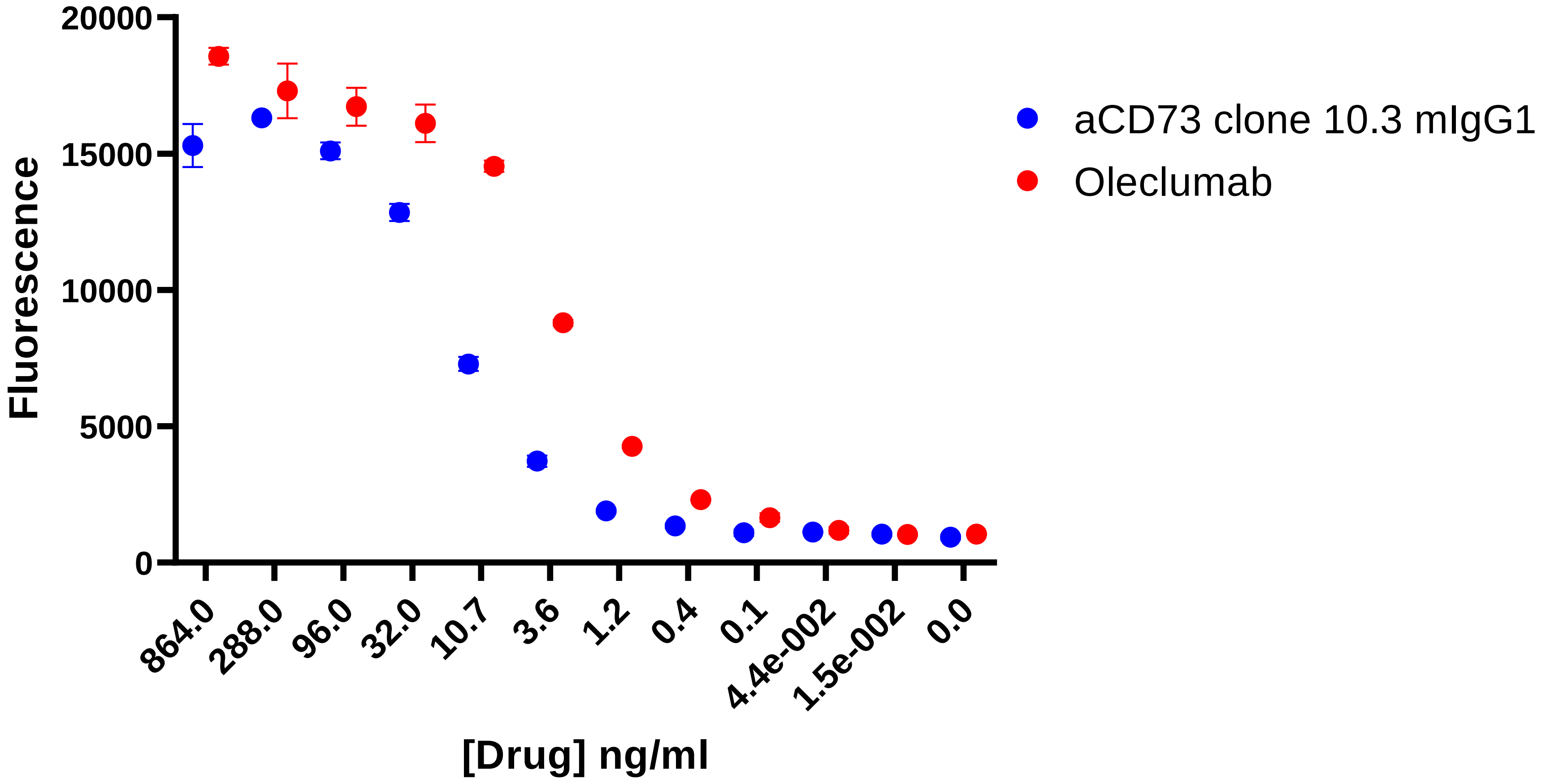

Supplemental Table-1 Details of antibodies used for IMC

Label	Clone	Channel	Dilution	Vendor	Product Code
**αSMA**	Polyclonal	Pr(141)	1:100	Standard BioTools	3141017D
**Vimentin**	D21H3	Nd(143)	1:100	Standard BioTools	3143027D
**CD68**	FA-11	Nd(145)	1:100	Bio Rad antibodies	MCA1957GA
**F4/80**	CI:A3-1	Gd(155)	1:100	Cell signalling	#70076
**CD163**	TNKUPJ	Gd(156)	1:50	Thermo Fisher	14-1631-82
**CD31**	390	Dy(164)	1:100	Thermo Fisher	14-0311-82
**CD206**	CD68C2	Tm(169)	1:50	Standard BioTools	3169021B
**DNA**	Intercalator	Ir(191)	1:400	Standard BioTools	201192A
**CollagenIV**	Polyclonal	Bi(209)	1:100	Novotec Labs	20451

Supplemental Table-2 IO monotherapies exert minimal transcriptomic changes, but chemotherapy and IO + IO / Chemo + IO combinations significantly perturb CT26 transcriptome

<u>DE cutoffs:</u>
Abs(log2FC) >=1
Adj-pval < 0.05

	**# of DE genes**	Gene Ontology (BP) enrichments (adjusted-pval < 0.05)	KEGG pathway enrichments (adjusted-pval < 0.05)
aPDL1	No DE genes	-	-
aCD73	1 DE genes:<br>1 Up-regulated	-	-
FF	435 DE genes:<br>277 Up-regulated & 158 Down-regulated	Immune response, Lymphocyte activation, Leukocyte activation, T cell activation, Interferon-gamma production	Natural killer cell mediated cytotoxicity<br>T cell receptor signaling pathway<br>Th1 and Th2 cell differentiation<br>Cytokine-cytokine receptor interaction
aCD73.aPDL1	1236 DE genes:<br>1049 Up-regulated & 187 Down-regulated	Inflammatory response, Myeloid leukocyte migration, Cell chemotaxis	Cytokine-cytokine receptor interaction<br>Hematopoietic cell lineage<br>Chemokine signaling pathway<br>Complement and coagulation cascades
FF.aCD73	964 DE genes:<br>902 Up-regulated & 62 Down-regulated	Immune response, Lymphocyte activation, Leukocyte activation, T cell activation, Interferon-gamma production	Cytokine-cytokine receptor interaction<br>Hematopoietic cell lineage<br>Chemokine signaling pathway<br>T cell receptor signaling pathway<br>Th1 and Th2 cell differentiation<br>NF-kappa B signaling pathway<br>Complement and coagulation cascades
FF.aPDL1	457 DE genes:<br>333 Up-regulated & 124 Down-regulated	Immune response, Lymphocyte activation, Leukocyte activation, T cell activation, Interferon-gamma production	T cell receptor signaling pathway<br>Cytokine-cytokine receptor interaction<br>Th1 and Th2 cell differentiation<br>Natural killer cell mediated cytotoxicity
FF.aCD73.aPDL1	1618 DE genes:<br>1490 Up-regulated & 128 Down-regulated	Immune response, Leukocyte activation, T cell activation, Lymphocyte activation	T cell receptor signaling pathway, Th1 and Th2 cell differentiation, Complement and coagulation cascades, Chemokine signaling pathway, Natural killer cell mediated cytotoxicity

Legends to Supplementary Figures and Tables:

Supp. Figure-S1 Caption: Responses in syngeneic mouse models to aCD73 and aPD-L1 treatments alone or in combination. BALB/c (CT26 cells in PBS, **A.**) and C57BL/6J mice (MCA205 cells in 50% Matrigel + PBS, **B.**) were implanted with 500,000 cells in the right flank and treated as shown in schematic in Figure-1A. Growth curves were plotted from calliper measurements done thrice weekly. aCD73 monotherapy didn't show any effect compared to control treated mice in both CT26 and MCA205 models. aPD-L1 monotherapy had extremely modest response in CT26 (1/13 CR) only. Combined treatment with aCD73 and aPD-L1 also didn't reveal any enhance response rate, with 1/13 CR mice observed in each CT26 and MCA205 tumor models. Average (±SEM) tumor volume data for CT26 model (**C, left panel**) and for MCA205 tumor model (**D, right panel**).

Supp. Figure-S1 Alt-text: A. Four panels of line graphs showing the individual response to different treatments in CT26 tumor model. B. Four panels of line graphs showing the individual response to different treatments in MCA205 tumor model. C. Two panels (upper and lower) of line graphs showing the average response to different treatments in CT26 tumor model. D. Two panels (upper and lower) of line graphs showing the average response to different treatments in MCA205 tumor model.

Supp. Figure-S2 Caption: Addition of aCD73 to 5FU+OHP and Docetaxel does not lead to enhanced cytotoxicity in vitro. 10,000 cells of each HCT-116, HT-29, CT26 and MCA-205 cells were seeded in 96 well plate and treated with serially diluted chemotherapeutics as indicated along with aCD73. Cytotoxicity was measured by CellTiter-Glo® Luminescent assay after 72 hours of incubation with drugs. As shown in different panels of results, none of the cell lines tested showed any additivity of aCD73 to either 5FU+OHP and Docetaxel.

Supp. Figure-S2 Alt-text: Five panels of dose response graphs in different cell lines, named on top of panel, showing that anti-CD73 treatment doesn't add to chemotherapy effects in these in vitro settings. Red line is the chemotherapy component, blue line is combination, green line is anti-CD73 treatment alone and black is untreated control.

Supp. Figure-S3 Caption: Addition of aCD73 and aPD-L1 to 5FU+OHP increases IFNg secreting CD8 and NK cells. (A) Schematic of the experimental design. (B) Flow cytometric analysis revealed increased frequencies of the interferon-gamma producing CD8 and NK cells in the group of mice treated with all aCD73 and aPD-L1 to 5FU+OHP compared to te chemotherapy alone or the aCD73+ 5FU+OHP or the aPD_L1 + 5FU+OHP groups.

Supp. Figure-S3 Alt-text: A. Experiment design and dosing scheme color coded to drug(s) being dosed. B Graph showing the percentage of interferon gamma secreting CD8 or NK cells in response to different treatments or their combinations in CT26 tumor model.

Supp. Figure-S4 Caption: Mass Spectrometry Imaging (MSI) confirms adenosine pathway modulation by addition of aCD73 to 5FU+OHP. (A) Schematic of the adenosine generation pathway. (B) MSI images showing the abundance of ATP as well different metabolites of the adenosine pathway in the CT26 tumours. 5FU+OHP lead to modest increase in ATP and AMP abundance compared to control treated tumours however addition of aCD73 to 5FU+OHP resulted in much decreased adenosine as well inosine and xanthine.

Supp. Figure-S4 Alt-text: A. Flow chart of adenosine generation pathway. B. A total of 15 images in three rows representing treatments and five columns representing important metabolites in the adenosine generation pathway. Images show modulation of the adenosine generation pathway by addition of anti-CD73 to the chemotherapy.

Supp. Figure-S5 Caption: Imaging Mass Cytometry showing the pharmacodynamic changes observed by addition of aCD73 to 5FU+OHP. IMC images of the CT26 tumors bearing mice treated with either control, 5FU+OHP and aCD73+5FU+OHP. aCD73+5FU+OHP combination treated mice tumors presented lower frequencies of cells expressing macrophage markers like CD68, F4/80 as well as suppressive tumor associated macrophage markers like CD163 and CD206 [left panel] as well as markers known to associate with cancer associated fibroblasts like collage-iv, alpha smooth muscle actin, vimentin along with CD31.

Supp. Figure-S5 Alt-text: CT26 tumor images with different markers for cell types showing changes seen in those markers in different treatment groups. Treatment groups are in the rows and cellular markers tested are in the columns.

Supp. Figure S6 Caption. Significant effects of treatments on top ranked gene signatures from Molecular Signatures Database (MSigDB). 5FU+OHP drives type 1 & 2 IFN response. Adding anti-CD73 to 5FU+OHP drives up inflammation and STAT5 pathway activation (IL-2). IO doublet drives TNF-a signaling, but not IFN-1s. 5FU+OHP+aCD73+aPD-L1 drives all of the above.

Supp. Figure-S6 Alt-text: Heatmap showing the profound changes seen in some of the immune related pathways using the database of Molecular Signatures.

Supp. Figure S7 Caption. Top 50 differentially expressed genes for Triple vs Control were identified and log_2 fold changes shown for different comparisons. Heatmap on the right panel shows expression change for selected immune-related genes across different conditions.

Supp. Figure S7 Alt-text: Left panel showing heatmap with changes seen in to 50 genes in RNAseq data, red color shows genes upregulated where blue color shows genes downregulated in relation to the comparator treatments. Right panel shows heatmap of the genes of most interest.

Supp. Figure-S8 Caption: Binding analysis of aCD73 clone 10.3 mIgG1 and oleclumab to human CD73. Binding ability of oleclumab and aCD73 10.3 mIgG1 to human CD73 was tested in an ELISA. Human CD73 was immobilised on the plate surface, blocked, and a dilution of aCD73 clone10.3 mIgG1 or oleclumab added to generate the binding curve. A HRP conjugated secondary was used to detect the fluorescent signal as plotted; red= oleclumab and blue= aCD73 10.3 mIgG1.

Supp. Figure-S8 Alt-text: Graph showing binding ability of oleclumab and aCD73 10.3 mIgG1 to human CD73 was tested in an ELISA using a dilution of aCD73 clone10.3 mIgG1 or oleclumab as plotted; red= oleclumab and blue= aCD73 10.3 mIgG1.

Supp. Table-1 Caption. Antibodies used for IMC experiment. The table shows the details of the antibodies used in IMC along with their clone, channel and dilution information.

Supp. Table-1 Alt-text: Table showing details of the antibodies used in imaging mass cytometry experiment.

Supp. Table-2 Caption. Chemotherapy, IO+IO and Chemo+IO combinations significantly perturb the CT26 transcriptome. Number of differentially expressed genes are shown for comparison of each condition to Control. Top ranked Gene Ontology Biological process and KEGG pathway enrichments for differentially up-regulated genes are listed.

Supp. Table-2 Alt-text: Table showing that monotherapy treatment of immuno-oncology drugs leads to minimal changes in genes however, chemotherapy, chemotherapy+IO agents as well as IO doublet treatments lead to a much larger

changes in genes expression. Rows in this table show different treatments, first column show number of genes perturbed and second and third column show the pathways affected.
